# The role of acid-base imbalance in statin-induced myotoxicity

**DOI:** 10.1016/j.trsl.2016.03.015

**Published:** 2016-08

**Authors:** Dhiaa A. Taha, Cornelia H. De Moor, David A. Barrett, Jong Bong Lee, Raj D. Gandhi, Chee Wei Hoo, Pavel Gershkovich

**Affiliations:** a Division of Medicinal Chemistry and Structural Biology, School of Pharmacy, University of Nottingham, Nottingham, UK; b Division of Molecular and Cellular Science, School of Pharmacy, University of Nottingham, Nottingham, UK

**Keywords:** cDNA, complementary DNA, Ct, cycle threshold, DMEM, Dulbecco's modified eagle medium, *Gapdh*, glyceraldehyde-3-phosphate dehydrogenase, *Hprt*, hypoxanthine phosphoribosyl transferase, HQC, high concentration quality control, IS, internal standard, LDH, lactate dehydrogenase, LLOQ, lower limit of quantification, LOV-A, lovastatin hydroxy acid, LOV-L, lovastatin lactone, LQC, low concentration quality control, *MHC*, myosin heavy chain, MQC, medium concentration quality control, mRNA, messenger RNA, *MRP*, multiresistant protein, MTT, thiazolyl blue tetrazolium bromide, NA, nonapplicable, *OATP*, organic anionic transporting polypeptide, PBS, phosphate buffer saline, PVA, pravastatin hydroxy acid, PVL, pravastatin lactone, RSD, relative standard deviation, RE, relative error, *Rps12*, ribosomal protein S12, SVA, simvastatin hydroxy acid, SVL, simvastatin lactone, *Tbp*, TATA box-binding protein

## Abstract

Disturbances in acid-base balance, such as acidosis and alkalosis, have potential to alter the pharmacologic and toxicologic outcomes of statin therapy. Statins are commonly prescribed for elderly patients who have multiple comorbidities such as diabetes mellitus, cardiovascular, and renal diseases. These patients are at risk of developing acid-base imbalance. In the present study, the effect of disturbances in acid-base balance on the interconversion of simvastatin and pravastatin between lactone and hydroxy acid forms have been investigated in physiological buffers, human plasma, and cell culture medium over pH ranging from 6.8–7.8. The effects of such interconversion on cellular uptake and myotoxicity of statins were assessed in vitro using C2C12 skeletal muscle cells under conditions relevant to acidosis, alkalosis, and physiological pH. Results indicate that the conversion of the lactone forms of simvastatin and pravastatin to the corresponding hydroxy acid is strongly pH dependent. At physiological and alkaline pH, substantial proportions of simvastatin lactone (SVL; ∼87% and 99%, respectively) and pravastatin lactone (PVL; ∼98% and 99%, respectively) were converted to the active hydroxy acid forms after 24 hours of incubation at 37°C. At acidic pH, conversion occurs to a lower extent, resulting in greater proportion of statin remaining in the more lipophilic lactone form. However, pH alteration did not influence the conversion of the hydroxy acid forms of simvastatin and pravastatin to the corresponding lactones. Furthermore, acidosis has been shown to hinder the metabolism of the lactone form of statins by inhibiting hepatic microsomal enzyme activities. Lipophilic SVL was found to be more cytotoxic to undifferentiated and differentiated skeletal muscle cells compared with more hydrophilic simvastatin hydroxy acid, PVL, and pravastatin hydroxy acid. Enhanced cytotoxicity of statins was observed under acidic conditions and is attributed to increased cellular uptake of the more lipophilic lactone or unionized hydroxy acid form. Consequently, our results suggest that comorbidities associated with acid-base imbalance can play a substantial role in the development and potentiation of statin-induced myotoxicity.

**At a glance commentary**

Taha DA, et al.

**Background**

Statins are commonly prescribed for elderly patients who are at risk of developing acid-base imbalance as a result of multiple comorbidities such as diabetes mellitus, cardiovascular, and renal diseases. Disturbances in acid-base balance, such as acidosis and alkalosis, have potential to affect the ratio between the lactone and acid forms of statins and alter the pharmacologic and toxicologic outcomes of statin therapy.

**Translational Significance**

This work provides a novel translational insight into the role of disturbances in acid-base balance in development of statin-induced muscle toxicity. The effect of acidosis on statin-induced muscle toxicity is particularly important in case of lipophilic statins, such as simvastatin. Enhanced cytotoxicity of statins was observed under acidic conditions as a result of increased cellular uptake of the more lipophilic lactone form or unionized hydroxy acid form. On the other hand, alkaline conditions were found to have a protective effect against statin-induced myotoxicity because of inability of statin to achieve adequate intracellular concentrations as a result of conversion to the more hydrophilic ionized hydroxy acid form.

## Introduction

Statins are cholesterol-lowering drugs commonly used to reduce morbidity and mortality associated with atherosclerotic cardiovascular diseases.^1^ Recent data from the National Center for Health Statistics reveals that 27.9% of American men and women of 40 years and older are taking statins.^2^ In recent years, a significant increase in the number of statin prescriptions has been reported by the British Heart Foundation with more than 7 million people currently taking either prescribed or over the counter statins in the UK.^3^ According to the American College of Cardiology and American Heart Association guidelines^4^, ^5^ for prediction of cardiovascular risk factors, more than 1 billion people worldwide are now estimated to use statins.^6^

Statins are generally well tolerated, but muscular adverse effects considerably influence drug tolerability and patient adherence especially with long-term use.^1^ The exact mechanism by which these drugs induce their myotoxic effects is not fully understood. Simvastatin, a highly lipophilic statin, is the most commonly prescribed cholesterol-lowering medication, and 42% of American adults who are using cholesterol-lowering drugs are prescribed this drug.^2^ It has been postulated that lipophilic statins are more myotoxic than hydrophilic ones, most probably because of their ability to penetrate skeletal muscle tissues and alter membrane structure.^7^, ^8^, ^9^ Nonetheless, the ability of lipophilic statins to penetrate hepatic cells makes them more potent in reducing elevated cholesterol levels.^9^ This property might explain their wider use comparing to hydrophilic statins.

**Fig 1:**
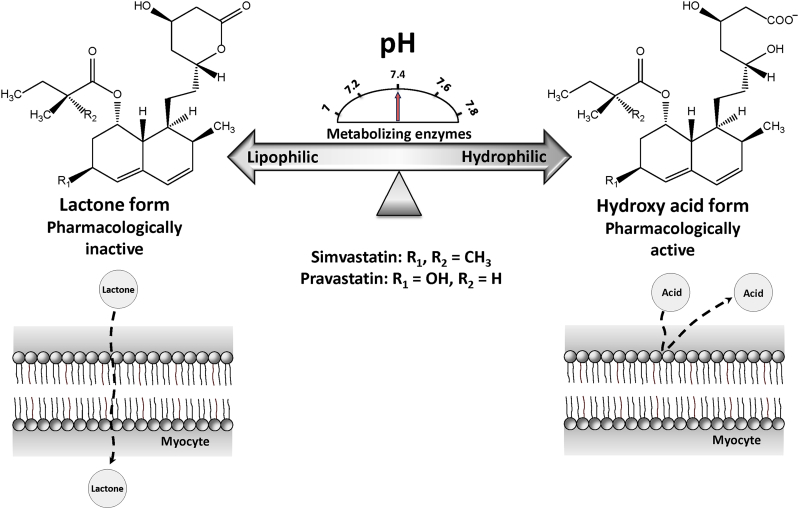
Schematic diagram for the possible mechanisms of statin interconversion between lactone and hydroxy acid forms and potential effect on membrane permeability. The interconversion between the 2 forms is mediated by pH and enzyme-dependent process. The higher lipophilicity of statins in the lactone form can potentially facilitate their penetration into muscle cells and consequently induce high local drug concentrations within skeletal muscle tissues.

Several risk factors have been suggested to predispose patients to statin-associated myotoxicity including advanced age, high dose, female gender, drug interactions, genetic variability of drug metabolizing enzymes and transporters, lipophilicity of statins, and coincident morbidities.^10^ Statins are administered either as lactone or hydroxy acid forms. The lactone form is pharmacologically inactive, whereas the hydroxy acid is the active form that lowers plasma cholesterol levels.^8^ Substantial differences exist between these forms in term of their lipophilicity. The lactone form is highly lipophilic, whereas hydroxy acid has poor lipid solubility.^9^, ^11^ It has been reported that lactone form is more myotoxic than the active acid form owing to its lipophilicity.^8^, ^12^, ^13^ In vivo, interconversion between both forms is mediated by enzymatic as well as pH-dependent chemical reaction in plasma, liver, and other tissues.^14^, ^15^, ^16^, ^17^ Therefore, acid-base imbalance can potentially alter the lipophilicity of statins by affecting their interconversion between lactone and hydroxy acid forms. The higher lipophilicity of statins in the lactone form can potentially facilitate their penetration into muscle cells and consequently induce high local drug concentrations within skeletal muscle tissues (Fig 1).

Disturbance in acid-base imbalance is quite common among statin users. Many patients receiving statins are elderly and have multiple co-morbidities such as diabetes mellitus, cardiovascular and renal diseases, or using diuretics. These conditions have been reported to lead to development of acidosis or alkalosis.^18^ Normally, the pH of blood plasma is maintained within narrow limits of 7.35–7.45. Most often, acidosis or alkalosis develops when there is a mild disturbance in blood plasma pH outside this range.^19^ Nevertheless, extreme disturbances in acid-base balance with blood pH of less than 7 or greater than 7.65 have been reported in intensive care units among patients with long-term diuretic abuse,^20^ diabetic ketoacidosis,^21^, ^22^ lactic acidosis,^23^, ^24^ hypovolemic shock,^25^ and ethylene glycol intoxication.^26^, ^27^, ^28^

Although acid-base imbalance has been proposed to be a possible risk factor for statin-induced myotoxicity, the effect of disturbances in acid-base balance on the development and potentiation of statin-related myotoxicity has received only limited investigation.^29^, ^30^, ^31^, ^32^ Limited numbers of in vitro studies have been done to evaluate the myotoxicity pattern of statins at pH relevant to acidosis and alkalosis using number of skeletal muscle cell lines. Results from these studies indicate enhanced cytotoxicity of statins under acidic conditions. However, the relationship between the pH-dependent interconversion of statins and their myotoxicity has not been addressed as a possible cause in these studies.^29^, ^30^, ^31^, ^32^

Therefore, the overall aim of this work was to elucidate the role of acid-base imbalance in statin-induced muscle toxicity. Simvastatin was selected as a model lipophilic statin in this study because of its high lipophilicity, wide clinical use, and high incidence of reported simvastatin-associated muscle toxicity.^2^, ^33^ Pravastatin was selected as a model hydrophilic statin with expected lower myotoxicity. The objectives included elucidation of the effect of disturbances in acid-base balance on the interconversion of statins between lactone and hydroxy acid forms, assessment of statin uptake by muscle cells under conditions relevant to acidosis, alkalosis and physiological pH, and evaluation of the role of statin interconversion in skeletal muscle toxicity.

## Materials and Methods

### Materials

Simvastatin lactone (SVL, 99.3%) and pravastatin hydroxy acid sodium (PVA, 99.4%) were purchased from Kemprotec Ltd (Lancashire, UK), simvastatin hydroxy acid ammonium salt (SVA, 98.0%) and pravastatin lactone (PVL, 98%) from Toronto Research Chemicals Inc (Toronto, Canada), lovastatin hydroxy acid sodium (LOV-A, 98.0%) and griseofulvin (97.0%) from Alfa-Aesar (Lancashire, UK), lovastatin lactone (LOV-L, 97.0%) from Cayman (Leicestershire, UK), and 4,4-dichlorodiphenyltrichloroethane (98.0%) from Sigma–Aldrich (Dorset, UK). Dulbecco's Modified Eagle Medium (42430025-Dulbecco's modified eagle medium [DMEM], high glucose, 4-2-hydroxyethyl-1-piperazineethanesulfonic acid without sodium pyruvate) was purchased from Invitrogen-Life Technologies (Paisley, UK). Thiazolyl blue tetrazolium bromide (MTT) was purchased from Alfa-Aesar (Lancashire, UK). Total RNA isolation kit (NucleoSpin RNA II) was supplied by Macherey-Nagel, GmbH & Co KG (Düren, Germany). GoTaq qPCR Master Mix was obtained from Promega (Southampton, UK). SuperScript IV Reverse Transcriptase was purchased from Invitrogen-Life Technologies (Paisley, UK). Random Hexamers (50 μM), dNTP mix (10 mM each), and Pierce lactate dehydrogenase (LDH) Cytotoxicity Assay kit were purchased from Thermo Fisher Scientific (Loughborough, UK). Pooled human liver microsomes of 20 mg/mL were purchased from Invitrogen-Life Technologies (Paisley, UK). MgCl_2_, KH_2_PO_4_, K_2_HPO_4_, and reduced nicotinamide adenine dinucleotide phosphate (NADPH) were purchased from Sigma–Aldrich (Gillingham, UK). All reagents used were of high performance liquid chromatography (HPLC) grade. Ultrapure water was obtained by passing distilled water through ELGA water purification system before use.

### The pH modification of human plasma, DMEM culture medium, and phosphate buffer saline

Sodium phosphate buffer has been used to adjust the pH of human plasma as previously described with some modification.^34^ The pH adjustment was done in 3 steps. In the first step, a fixed volume of human plasma samples was adjusted to the target pH (6.8–7.8) by adding appropriate volumes of either mono or dibasic sodium phosphate solution. After that, series of sodium phosphate buffer solutions of predefined pH were prepared at a concentration of 1 mol/L by mixing appropriate volumes of mono and dibasic sodium phosphates solution. Finally, one volume of phosphate buffer solutions (of defined pH) was mixed with 9 volumes of plasma samples, the pH of the resulting mixture was measured by Mettler Toledo T50 pH titrator, and minor adjustments were made as appropriate.

The pH of DMEM culture medium was adjusted to the target values (6.8–7.8) by adding an appropriate volume of either 1.0 N HCl or NaOH to a medium containing 20 mmol/L 4-2-hydroxyethyl-1-piperazineethanesulfonic acid (HEPES) buffer. The pH was measured at incubation temperature (37°C) by Mettler Toledo T50 pH titrator. Before the experiments, the medium was kept in an incubator for 24 hours under cell culture conditions (37°C and 5% CO_2_) to allow the desired pH ranges to equilibrate. After equilibration, minor adjustment in pH was occasionally required to reach the desired final pH.^35^ Maintaining medium under constant CO_2_ environment of 5% stabilizes the pH through CO_2_-HCO_3_^−^ equilibrium.^36^ The pH of phosphate buffer saline (PBS) was adjusted to the target values (6.8–7.8) in the same way without the need for equilibration.

### Interconversion of statins in human plasma, PBS, and DMEM culture medium

The interconversion of the lactone and hydroxy acid forms of simvastatin and pravastatin was investigated by incubating tested compounds with human plasma, PBS, and DMEM culture medium of defined pH values at 37°C for a predetermined period of time. Four different concentrations were examined for each statin (12.5, 25, 50, and 100 μmol/L for SVL and SVA and 25, 50, 100, and 200 μmol/L for PVL and PVA). Human plasma and PBS samples were incubated in C25 classic incubator shaker (New Brunswick Scientific). For DMEM samples, CO_2_ supply was maintained at 5% throughout experiment in a tissue culture incubator. The pH of different matrices was adjusted to simulate physiological pH, acidosis, and alkalosis as described previously. After incubation, the interconversion reaction was stopped at predetermined time points by adding 50 μL of ice-cold ammonium acetate buffer (100 mmol/L, pH 4.5) to 100 μL of tested samples.^14^ Six replicates were tested at each pH level, and the concentrations of parent and corresponding forms were measured by fully validated HPLC analytical methods.

### Metabolic microsomal stability

Microsomal metabolic stability assay was conducted using human liver microsomes. The reaction mixture consisted of MgCl_2_, human liver microsomes, NADPH, and SVL at final concentrations of 10 mmol/L, 0.5 mg protein/mL, 1 mmol/L, and 1 μmol/L, respectively, in 84.7 mmol/L potassium phosphate buffer. The buffer was prepared at 3 different pH levels of 6.8, 7.4, and 7.8 (simulating acidosis, physiological pH, and alkalosis, respectively). The reaction mixture was preincubated at 37°C in a water bath for 3 minutes before the assay. The reaction was initiated by the addition of NADPH to the mixture. Samples of 100 μL volume were withdrawn from the reaction mixture at 0.25, 5, 10, 20, 30, and 40 minutes and transferred into new tubes containing 1 mL of acetonitrile (ACN) to terminate the reaction. Vortex mixing of 30 seconds was applied to each sample, and then, the samples were subjected to sample preparation for liquid chromatography-tandem mass spectrometry (LC MS/MS) analysis. Half-life (*t*_*1/2*_) of SVL was calculated using the following equation:

(Eq. 1) $$t_{1/2}=-\frac{0.693}{k}$$

where *k* is the slope from the plot of natural log percentage of SVL versus incubation time. The experiment was performed in triplicates.

### C2C12 growth and differentiation

C2C12 mouse myoblast cells were cultured in a humidified environment of 5% CO_2_ at 37°C. Cells were maintained subconfluent (70%–80%) by growing in DMEM medium supplemented with 10% fetal bovine serum, 1% L-glutamine, and 1% penicillin-streptomycin antibiotic mixture. Myogenic differentiation was induced by growing the cells in differentiation medium containing 2% horse serum. The cells were cultured over a period of 4–6 days to allow complete differentiation, and the medium was replaced every 24 hours.^37^

To verify the differentiation of C2C12, the expression of 2 markers of myogenic differentiation (myogenin and myosin heavy chain [*MHC*]) were assessed by real-time reverse transcriptase quantitative polymerase chain reaction (qPCR) at different stage of cells differentiation. C2C12 was cultured on a 6-well plate at a density of 2 × 10^5^/mL. The cells were allowed to attach to the well surface by incubation at 37°C and 5% CO_2_. Differentiation was induced on 80% confluence by switching the cells to differentiation medium. On differentiation, the medium was aspirated from the wells, and the cells were harvested. Total RNA was extracted from undifferentiated and differentiated cells at different differentiation stages (day 1, 3, 5, 7, and 9) using NucleoSpin RNA II extraction kit (the manufacturer's protocol was followed). The extracted RNA was reverse transcribed into complementary DNA (cDNA) using random hexamer primers, dNTP mix (10 mM each), and SuperScript VI (reverse transcriptase purified from *Escherichia. coli*), and the resultant cDNA was used as a template for polymerase chain reaction (PCR) amplification.

The expression of differentiation marker genes (myogenin and *MHC*) and 4 reference genes (TATA box-binding protein [*Tbp*], glyceraldehyde-3-phosphate dehydrogenase [*Gapdh*], hypoxanthine-guanine phosphoribosyltransferase [*Hprt*], and ribosomal protein S12 [*Rps12*]) was assessed by real-time qPCR using GoTaq qPCR Master Mix (Promega, UK) and previously published primer sets.^38^ The primers used for the qPCR were synthesized by Sigma–Aldrich Biotechnology, UK (sequences are listed in Supplementary Table I).

DNA amplification was carried out using Rotor-Gene Q, (Qiagen thermal cycler). The PCR thermocycling program consisted of an initial denaturation step at 95°C for 10 minutes, followed by 45 cycles of 30 seconds at 95°C, 30 seconds at the optimal annealing temperature of 55°C, and 30 seconds at 72°C for extension. To confirm the amplification specificity, PCR products were subjected to melting curve analysis. A standard curve was constructed for each sample and was derived from 10-fold serial dilution of cDNA template.

The levels of gene expressions in each sample over the days of differentiation were calculated relative to their expression in undifferentiated cells. Data were normalized relative to the expression of *Tbp* gene (a reference gene with minimal fluctuation in expression over the periods of cell differentiation). All samples were run in triplicate, and the mean value was used for subsequent analysis. The ΔΔcycle threshold (Ct) value for each gene was determined by calculating the difference between the Ct value of the target gene and the Ct value of the reference gene. The normalized level of the gene expression in each sample was calculated using the formula 2^−ΔΔCt^, and the results were expressed as fold changes in gene expression from the baseline level observed with undifferentiated C2C12 myoblast cells. Three reference genes (*Gapdh*, *Hprt*, and *Rps12*) were used for validation of gene expression. The size and the integrity of the amplicon in each sample was assessed by running the qPCR products on 1.2% agarose gel after staining with SYBR safe (Invitrogen-Life Technologies, UK), and the bands were visualized under UV light.

### Cellular uptake of statins in response to altered medium pH

C2C12 cells were cultured in 10 cm dishes at a density of 2 × 10^5^/mL. Cells were allowed to attach for 24 hours or to differentiate for 4 days by switching to differentiation medium containing 2% horse serum. Before starting the uptake study, the medium was removed and replaced by a fresh medium of modified pH (relevant to physiological pH, acidosis, and alkalosis) and the cells were allowed to equilibrate for 10 minutes. The uptake study was initiated by adding SVL, SVA, PVL, or PVA to medium at a concentration of 1 μmol/L. Cells were incubated for a predetermined period of time at 37°C under CO_2_ environment of 5%. Statins uptake was terminated by suctioning off the DMEM containing the tested drug, and the cells were washed twice with ice-cold PBS (pH 7.4). Cells were harvested by trypsinization and resuspended in 200 μL PBS (pH 7.4). Five replicates were tested at each pH level and the intracellular concentration of both the lactone and the corresponding hydroxy acid forms of simvastatin and pravastatin were determined in 100 μL of cell suspension using fully validated LC MS/MS assays. Cellular protein content was determined in the remaining cell suspension after pelleting and lysing the cells with radio-immunoprecipitation issay (RIPA) lysis buffer (150 mmol/L NaCl, 50 mmol/L Tris-HCl [pH 8.0], 0.5% Nonidet P-40 [NP-40], supplemented with proteinase inhibitor and phosphatase inhibitor), using Bradford protein assay and bovine serum albumin as a standard.^39^

### Analytical procedures

#### HPLC analysis

Fully validated HPLC-UV methods were used to determine the concentrations of the lactone and the corresponding hydroxy acid forms of simvastatin and pravastatin after interconversion studies in human plasma, PBS, and DMEM culture medium of different pH. Samples for HPLC analysis were prepared by protein precipitation followed by liquid-liquid extraction. A 100 μL of human plasma or DMEM samples were pipetted into 1.5 mL Eppendorf tubes, and 50 μL of ammonium acetate buffer (100 mmol/L, pH 4.5) along with 10 μL of internal standard solution (4,4-dichlorodiphenyltrichloroethane [DDT] or griseofulvin 100 μg/mL for simvastatin and pravastatin containing samples, respectively) was added, and samples were vortex mixed for 30 seconds. Proteins were precipitated by the addition of 300 μL of chilled ACN, and samples were vortex mixed for 1 minute and then centrifuged at 900*g* for 10 minutes at 4°C. After centrifugation, the ACN layer was transferred into new glass test tubes, and 3 mL of methyl tert-butyl ether were added to each sample, vortex mixed for 10 minutes, and centrifuged at 1,615*g* for 10 minutes at 4°C. Finally, the upper organic layers were separated using glass Pasteur pipette, transferred into new glass tubes, and the contents of the tubes were evaporated to dryness using Techne Sample Concentrator (Bibby Scientific Ltd, UK). The dry residues were reconstituted with 100 μL of the mobile phase, vortex mixed, and placed into appropriate HPLC vials. Samples from PBS were extracted in the same way with one exception that the protein precipitation step was skipped.

HPLC analysis was performed using Waters Alliance 2965 separation module equipped with Waters 996 Photodiode Array Detector and integrated autosampler. System control and data processing were performed using Empower software. Chromatographic separation was achieved by ACE Excel Super C18 column (100 × 3 mm, 3 μm) under isocratic conditions with mobile phase consisting of ACN: 5 mmol/L ammonium acetate buffer, pH 4.5 (73:27 and 55:45, v/v for simvastatin and pravastatin samples, respectively). The flow rate was set at 0.4 mL/min for simvastatin and 0.3 mL/min for pravastatin samples. Samples temperature was kept at 4°C, and column temperature was set at 40°C. Chromatographic separation was monitored by photodiode array detector at 238 nm with an injection volume of 20 μL.

#### LC MS/MS analysis

The intracellular concentrations of the hydroxy acid and lactone forms of simvastatin and pravastatin were determined by LC MS/MS method. A 100 μL of cell suspension was transferred into 1.5 mL Eppendorf tubes, and 50 μL of ammonium acetate buffer (100 mmol/L, pH 4.5) along with 10 μL of internal standard solutions (LOV-A and LOV-L, 2.5 μg/mL) was added, and samples were vortex mixed for 30 seconds. Cell lysis was performed using Retsch MM-301 mixer mill after the addition of 300 μL of ice-cold ACN. Samples were centrifuged at 15,000*g* for 10 minutes at 4°C to remove cell debris. After centrifugation, the ACN layer was transferred into new glass test tubes, and liquid-liquid extraction was performed in the same way as described for HPLC-UV method using methyl tert-butyl ether.

LC MS/MS system consisted of Quattro Ultima triple-quadrupole mass spectrometer (Micromass, UK) interfaced via an electrospray ionization probe with Agilent (1100 Series, Agilent Technologies) HPLC system. The HPLC system consisted of binary pump, online degasser, temperature-controlled autosampler, and column compartment. Chromatographic separation was achieved by ACE Excel Super C18 column (100 × 3 mm, 3 μm) with mobile phase consisting of ACN: 5 mmol/L ammonium acetate buffer, pH 4.5. An isocratic condition consisted of ACN: ammonium acetate (80:20, v/v) was used for simvastatin separation. Pravastatin was separated using gradient flow as follows: 65% ACN over the first 4 minutes, increased to 75% ACN over a period of 1 minute and kept at 75% ACN for another 2 minutes, and then the flow was returned to 65% ACN in the last minute to prepare for the next run. The flow rate was set at 0.3 mL/min. Samples temperature was kept at 4°C, and column temperature was set at 40°C.

Quantification was performed using multiple-reaction monitoring scan. The mass spectrometric system was operated in the negative ionization mode for quantification of the hydroxy acid forms and positive mode for determination of the lactone forms. Instrument control and data acquisition were performed by MassLynx software packages (version 4.1). Data processing and analysis were performed using QuanLynx software. Nitrogen was used for nebulization and as a dissolution gas, whereas argon was used as a collision gas. Source temperature and desolvation temperature were setup at 125 and 350°C, respectively. The flow rate of the cone gas and desolvation gas was set at 150 and 565 L/h, respectively. Capillary voltage, cone voltage, and collision energy were optimized individually for each compound as indicated in Supplementary Table II by direct infusion with mobile phase using injection pump.

#### Analytical methods validation

Validation of the analytical methods was carried out by determining the intraday and interday accuracy and precision. Six replicates of statin containing samples (human plasma, PBS, DMEM, and cell lysate) were analyzed at 4 quality control (QC) levels (lower limit of quantification, low, medium, and high concentration quality control). Precision was expressed as relative standard deviation (%), whereas accuracy was described as relative error (%) and was determined by comparing the calculated concentration obtained using calibration curves to the theoretical concentrations. Intraday and interday precisions and accuracies were calculated by analyzing QC samples on the same day and on 6 different days over a period of 1 month, respectively.^40^, ^41^, ^42^ The acceptance criteria for intraday and interday precisions and accuracies were set at 15% for the high, medium, and low concentration QC samples and at 20% for lower limits of quantification.^40^, ^41^, ^42^

Extraction recoveries of the lactone and hydroxy acid forms of statins were also determined at high, medium, and low QC levels by comparing the peak area ratios of the analytes spiked after extraction to those spiked before extraction.^43^, ^44^, ^45^ Sex replicates were evaluated at each QC level.

### Expression of cellular transporters

The messenger RNA (mRNA) expression levels of organic anionic transporting polypeptide (*OATP*) *OATP1a4* and *OATP2b1* (uptake transporters) and multiresistant protein (*MRP*) *MRP1*, *MRP4*, and *MRP5* (efflux transporters) were assessed using reverse transcriptase PCR analysis. C2C12 cells were cultured in 10 cm dishes as described previously. Both undifferentiated and differentiated cells were maintained in their corresponding medium of modified pH (relevant to the physiological pH, acidosis, and alkalosis) for 6 hours. RNA extraction, cDNA synthesis, and gene amplifications were performed as described above in C2C12 growth and differentiation section. The primer sequences of the transporter and reference genes are listed in Supplementary Table III. Three different sets of primers were attempted to amplify *OATP1a4* and *OATP2b1* influx transporters. The levels of gene expressions in undifferentiated and differentiated cell samples maintained at different pH levels were calculated relative to their expression in undifferentiated cells maintained at physiological pH. Transporters genes were normalized to *Gapdh* whereas each reference gene was normalized relative to the other 3 reference genes. Results were expressed as fold changes in gene expression relative to the baseline level observed with undifferentiated C2C12 maintained at physiological pH.

### In vitro cytotoxicity of statins in medium with different pH levels

The effect of medium pH alteration on the cytotoxicity of statins was evaluated by MTT and LDH cytotoxicity assays. Both undifferentiated and differentiated C2C12 cells were used in these studies. For MTT assay, cells were seeded at a density of 4,000 cells/well in 96-well plates and allowed to attach for 24 hours or to differentiate for 4 days. Before starting treatment, the medium was replaced by a fresh one with modified pH (6.8, 7.4, and 7.8). C2C12 cells were treated with different concentrations of SVL, SVA, PVL, or PVA prepared in dimethyl sulfoxide (DMSO) for 72 hours. The final level of DMSO in culture medium was determined by DMSO tolerance study and was found to be 0.25% (v/v) with no obvious cytotoxicity (100% viability compared to untreated cells). Constant volume of DMSO was maintained for all samples, and DMSO alone without statins has been used as control. Positive control samples with 100% cell death were obtained by treating cells with 1% Triton X-100. Produced formazan crystals were dissolved in DMSO, and the absorbance was measured at 570 nm using EnVision Multilabel Plate Reader (PerkinElmer). Results were expressed as percentage of the control, and IC_50_ was calculated using prism 6 (GraphPad Software Inc).

For LDH assay, cells were cultured and treated as described previously, and the assay was performed according to manufacturer instructions. Formation of red formazan product was monitored at 490 nm with a reference wavelength of 680 nm. Positive control samples with 100% LDH release were obtained by fully lysing the cells before LDH assay to determine the maximum amount of LDH present in the cells. All assays were performed in triplicate, and data were expressed as the ratio of the amount of LDH released, per treatment, to the maximum amount of LDH released from the control cells.

### Statistical analysis

Data were expressed as mean ± SD. Statistical differences between groups were determined by one-way analysis of variance, 2-way analysis of variance, or Kruskal-Wallis test followed by Tukey's, Bonferroni's, or Dunn's test for multiple comparisons, as appropriate. A *P*-value of less than 0.05 was considered to be statistically significant.

## Results

### Analytical procedures

Representative chromatographies and validation data of analytical procedures are provided in Supplementary Materials.

### Interconversion of statins in human plasma, PBS, and DMEM culture medium

**Supplementary Fig 1:**
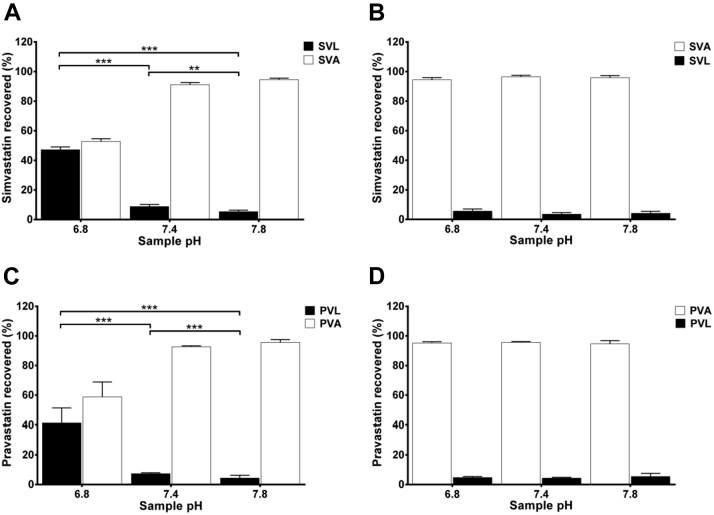
Interconversion of statins between lactone and hydroxy acid forms in phosphate buffer saline at different pH levels. Simvastatin lactone (**A**), simvastatin hydroxy acid (**B**), pravastatin lactone (**C**), and pravastatin hydroxy acid (**D**) were incubated with PBS of modified pH (6.8–7.8) at a concentration of 50 μmol/L for 24 hours at 37°C. The percentages of the lactone and hydroxy acid form recovered after 24 hours are expressed as mean ± standard deviation, (n = 6). Differences between samples of different pH were analyzed by one-way analysis of variance followed by Tukey's post hoc test (^∗∗∗^*P* < 0.001; **P < 0.01). SVL, simvastatin lactone; SVA, simvastatin hydroxy acid; PVL, pravastatin lactone; PVA, pravastatin hydroxy acid.

**Supplementary Fig 2:**
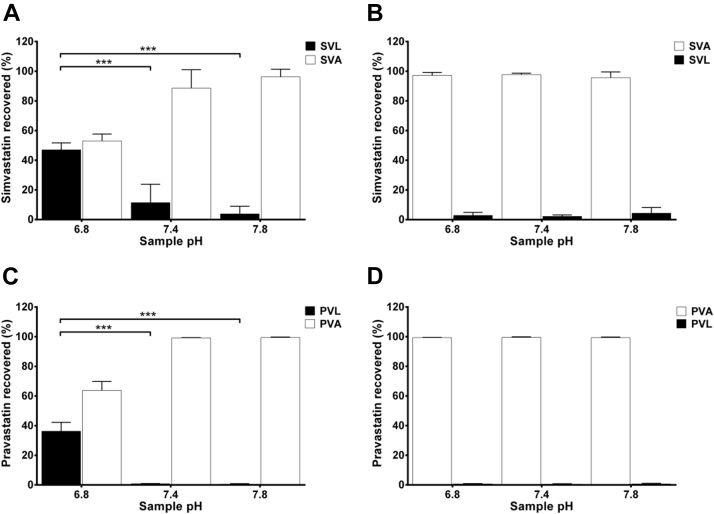
Interconversion of statins between lactone and hydroxy acid forms in Dulbecco's modified eagle medium culture medium at different pH levels. Simvastatin lactone (**A**), simvastatin hydroxy acid (**B**), pravastatin lactone (**C**), and pravastatin hydroxy acid (**D**) were incubated with Dulbecco's modified eagle medium culture medium of modified pH (6.8–7.8) at a concentration of 50 μmol/L for 24 hours at 37°C. The percentages of the lactone and hydroxy acid form recovered after 24 hours are expressed as mean ± standard deviation, (n = 6). Differences between samples of different pH were analyzed by one-way analysis of variance followed by Tukey's post hoc test (^∗∗∗^*P* < 0.001). SVL, simvastatin lactone; SVA, simvastatin hydroxy acid; PVL, pravastatin lactone; PVA, pravastatin hydroxy acid.

**Fig 2:**
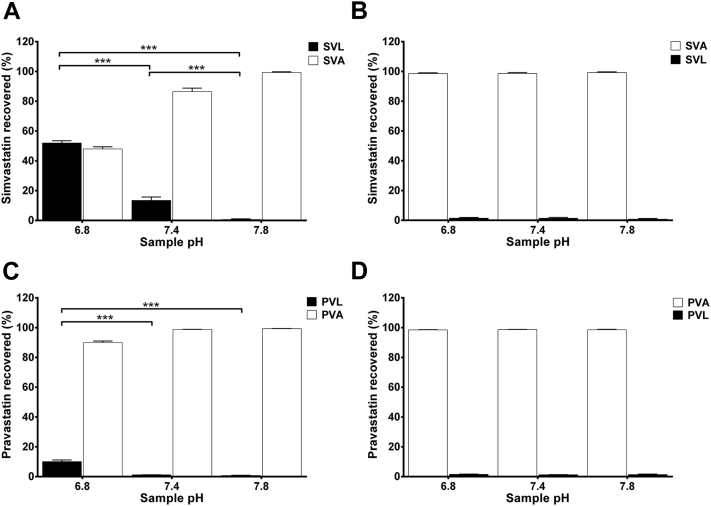
Interconversion of simvastatin and pravastatin between lactone and hydroxy acid forms in human plasma of different pH levels. Simvastatin lactone (**A**), simvastatin hydroxy acid (**B**), pravastatin lactone (**C**), and pravastatin hydroxy acid (**D**) were incubated with human plasma of modified pH (6.8–7.8) at a concentration of 50 μmol/L for 24 hours at 37°C. The percentages of lactone and hydroxy acid form recovered after 24 hours are expressed as mean ± standard deviation, (n = 6). Differences between samples of different pH were analyzed by one-way analysis of variance followed by Tukey's post hoc test (***P < 0.001). SVL, simvastatin lactone; SVA, simvastatin hydroxy acid; PVL, pravastatin lactone; PVA, pravastatin hydroxy acid.

To characterize the effect of pH alteration on interconversion of statins, the lactone and hydroxy acid forms of simvastatin and pravastatin were incubated with human plasma, PBS, and DMEM culture medium of pH relevant to acidosis, alkalosis, and physiological pH for 24 hours. Fig 2 shows the results of simvastatin and pravastatin interconversion between lactone and hydroxy acid forms in human plasma of different pH levels at a concentration of 50 μmol/L. Results indicate that the conversion of the lactone form of simvastatin and pravastatin to the corresponding hydroxy acid is strongly pH dependent. At physiological and alkaline pH, substantial proportions of SVL (∼87% and 99%, respectively) and PVL (∼98% and 99%, respectively) were converted to the active hydroxy acid forms after 24 hours of incubation at 37°C. At acidic pH, such conversion occurs to a lower extent, resulting in greater proportion of statins remaining in the more lipophilic lactone form (Fig 2, panel A and C). On the other hand, pH alteration has not been shown to influence the conversion of the hydroxy acid form to the corresponding lactone form (Fig 2, panel B and D). Results of statin interconversion between lactone and hydroxy acid forms in PBS and DMEM culture medium are shown in Supplementary Figs 1 and 2, respectively.

**Supplementary Fig 3:**
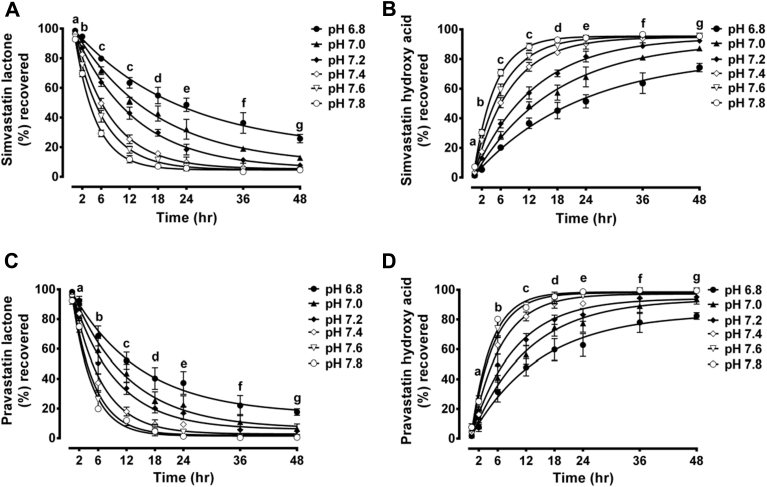
Time course interconversion of simvastatin lactone (SVL) and pravastatin lactone (PVL) in phosphate buffer saline at different pH levels. The lactone forms of simvastatin and pravastatin were incubated with phosphate buffer saline of modified pH (6.8–7.8) at a concentration of 50 μmol/L for 48 hours at 37°C. The percentages of lactone and hydroxy acid form recovered at different time points are expressed as mean ± standard deviation, (n = 6). Differences between samples of different pH were analyzed by 2-way analysis of variance followed by Bonferroni's post hoc test. Time course interconversion of SVL shows disappearance of SVL (**A**) and formation of simvastatin hydroxy acid form (**B**). a = pH 7.8 vs pH 6.8, 7.0 (*P* < 0.001); pH 7.8 vs pH 7.2 (*P* < 0.05). b = pH 6.8 vs pH 7.2, 7.4, 7.6, 7.8; pH 7.0 vs pH 7.2, 7.4, 7.6, 7.8; pH 7.2 vs pH 7.6, 7.8; pH 7.4 vs pH 7.6, 7.8; pH 7.6 vs pH 7.8 (*P* < 0.001); pH 7.2 vs pH 7.4 (*P* < 0.05). c = pH 6.8 vs pH 7.0, 7.2, 7.4, 7.6, 7.8; pH 7.0 vs pH 7.2, 7.4, 7.6, 7.8; pH 7.2 vs pH 7.4, 7.6, 7.8; pH 7.4 vs pH 7.6, 7.8; pH 7.6 vs pH 7.8 (*P* < 0.001). d = pH 6.8 vs pH 7.0, 7.2, 7.4, 7.6, 7.8; pH 7.0 vs pH 7.2, 7.4, 7.6, 7.8; pH 7.2 vs pH 7.4, 7.6, 7.8; pH 7.4 vs pH 7.8 (*P* < 0.001); pH 7.4 vs pH 7.6 (*P* < 0.01); pH 7.6 vs pH 7.8 (*P* < 0.05). e = pH 6.8 vs pH 7.0, 7.2, 7.4, 7.6, 7.8; pH 7.0 vs pH 7.2, 7.4, 7.6, 7.8; pH 7.2 vs pH 7.4, 7.6, 7.8 (*P* < 0.001); pH 7.4 vs pH 7.8 (*P* < 0.05). f = pH 6.8 vs pH 7.0, 7.2, 7.4, 7.6, 7.8; pH 7.0 vs pH 7.2, 7.4, 7.6, 7.8; pH 7.2 vs pH 7.4, 7.6, 7.8 (*P* < 0.001). g = pH 6.8 vs pH 7.0, 7.2, 7.4, 7.6, 7.8; pH 7.0 vs pH 7.2, 7.4, 7.6, 7.8 (*P* < 0.001). Time course interconversion of PVL shows disappearance of PVL (**C**) and formation of pravastatin hydroxy acid form (**D**). a = pH 6.8 vs pH 7.6, 7.8; pH 7.0 vs pH 7.6, 7.8; pH 7.2 vs pH 7.8 (*P* < 0.001); pH 7.4 vs pH 6.8, 7.8; pH 7.2 vs pH 7.6 (*P* < 0.01); pH 7.0 vs pH 7.4 (*P* < 0.05). b = pH 6.8 vs pH 7.0, 7.2, 7.4, 7.6, 7.8; pH 7.0 vs pH 7.4, 7.6, 7.8; pH 7.2 vs pH 7.4, 7.6, 7.8; pH 7.4 vs pH 7.6, 7.8 (*P* < 0.001); pH 7.0 vs pH 7.2 (*P* < 0.01). c = pH 6.8 vs pH 7.2, 7.4, 7.6, 7.8; pH 7.0 vs 7.2, 7.4, 7.6, 7.8; pH 7.2 vs pH 7.4, 7.6, 7.8 (*P* < 0.001); pH 6.8 vs pH 7.0 (*P* < 0.01); pH 7.4 vs pH 7.6 (*P* < 0.05). d = pH 6.8 vs pH 7.0, 7.2, 7.4, 7.6, 7.8; pH 7.0 vs pH 7.4, 7.6, 7.8; pH 7.2 vs pH 7.4, 7.6, 7.8 (*P* < 0.001). e = 6.8 vs pH 7.0, 7.2, 7.4, 7.6, 7.8; pH 7.0 vs pH 7.4, 7.6, 7.8; pH 7.2 vs pH 7.6, 7.8, (*P* < 0.001); pH 7.4 vs pH 7.8 (*P* < 0.01); pH 7.2 vs pH 7.4 (*P* < 0.05). f = pH 6.8 vs pH 7.0, 7.2, 7.4, 7.6, 7.8; pH 7.0 vs pH 7.4, 7.6, 7.8 (*P* < 0.001). g = pH 6.8 vs pH 7.0, 7.2, 7.4, 7.6, 7.8 (*P* < 0.001).

**Supplementary Fig 4:**
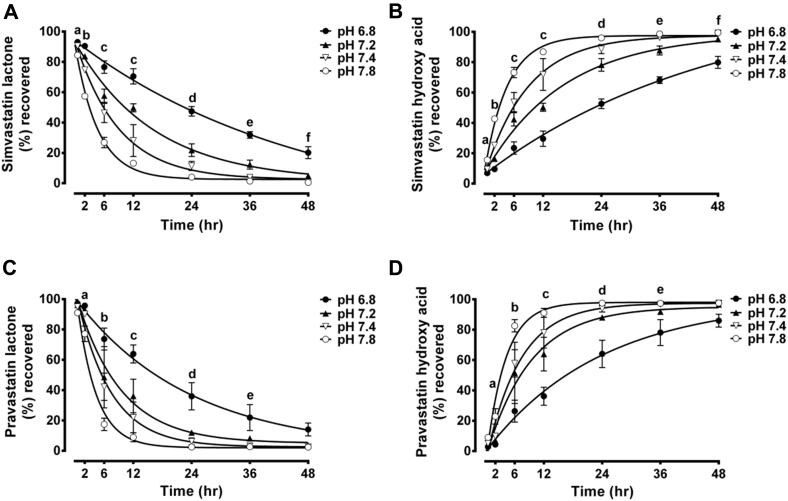
Time course interconversion of simvastatin lactone (SVL) and pravastatin lactone (PVL) in Dulbecco's modified eagle medium culture medium at different pH levels. The lactone forms of simvastatin and pravastatin were incubated with Dulbecco's modified eagle mediumculture medium of modified pH (6.8–7.8) at a concentration of 50 μmol/L for 48 hours at 37°C. The percentages of lactone and hydroxy acid form recovered at different time points are expressed as mean ± standard deviation, (n = 6). Differences between samples of different pH were analyzed by 2-way analysis of variance followed by Bonferroni's post hoc test. Time course interconversion of SVL shows disappearance of SVL (**A**) and formation of simvastatin hydroxy acid form (**B**). a = pH 7.8 vs pH 6.8, 7.2 (*P* < 0.01). b = pH 6.8 vs pH 7.2 (*P* < 0.05); pH 6.8 vs pH 7.4, 7.8; pH 7.2 vs pH 7.4, 7.8; pH 7.4 vs pH 7.8 (*P* < 0.001). c = pH 6.8 vs pH 7.2, 7.4, 7.8; pH 7.2 vs pH 7.4, 7.8; pH 7.4 vs 7.8 (*P* < 0.001). d = pH 6.8 vs pH 7.2, 7.4, 7.8; pH 7.2 vs pH 7.4, 7.8 (*P* < 0.001); pH 7.4 vs 7.8 (*P* < 0.01). e = pH 6.8 vs pH 7.2, 7.4, 7.8; pH 7.2 vs pH 7.4, 7.8 (*P* < 0.001). f = pH 6.8 vs pH 7.2, 7.4, 7.8 (*P* < 0.001). Time course interconversion of PVL shows disappearance of PVL (**C**) and formation of pravastatin hydroxy acid form (**D**). a = pH 6.8 vs pH 7.8 (*P* < 0.01); pH 7.2 vs pH 7.8 (*P* < 0.05). b = pH 6.8 vs pH 7.2, 7.4, 7.8; pH 7.8 vs pH 7.2, 7.4 (*P* < 0.001). c = 6.8 vs pH 7.2, 7.4, 7.8; pH 7.2 vs 7.8 (*P* < 0.001); pH 7.2 vs pH 7.4 (*P* < 0.05). d = pH 6.8 vs pH 7.2, 7.4, 7.8 (*P* < 0.001). e = pH 6.8 vs pH 7.4, 7.8 (*P* < 0.001); pH 6.8 vs pH 7.2 (*P* < 0.05).

**Fig 3:**
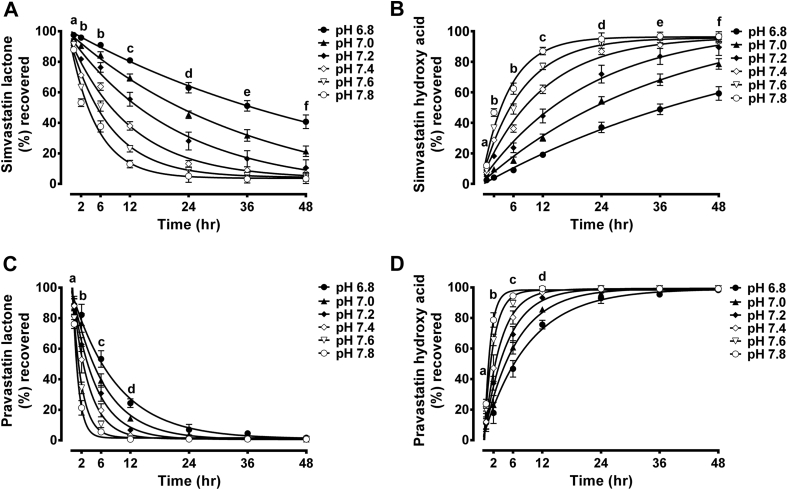
Time course interconversion of simvastatin lactone (SVL) and pravastatin lactone (PVL) in human plasma at different pH levels. The lactone forms of simvastatin and pravastatin were incubated with human plasma of modified pH (6.8–7.8) at a concentration of 50 μmol/L for 48 hours at 37°C. The percentages of lactone and hydroxy acid form recovered at different time points are expressed as mean ± standard deviation, (n = 6). Differences between samples of different pH were analyzed by 2-way analysis of variance followed by Bonferroni's post hoc test. Time course interconversion of SVL shows disappearance of SVL (**A**) and formation of simvastatin hydroxy acid form (**B**). a = pH 7.0 vs pH 7.6 (*P* < 0.05); pH 7.4 vs pH 6.8, 7.8 (*P* < 0.01); pH 6.8 vs pH 7.6, 7.8; pH 7.8 vs pH 7.0, 7.2 (*P* < 0.001). b = pH 6.8 vs pH 7.0, 7.2, 7.4, 7.6, 7.8; pH 7.0 vs pH 7.2, 7.4, 7.6, 7.8; pH 7.2 vs pH 7.4, 7.6, 7.8; pH 7.4 vs pH 7.6, 7.8; pH 7.6 vs pH 7.8 (*P* < 0.001). c = pH 6.8 vs pH 7.0, 7.2, 7.4, 7.6, 7.8; pH 7.0 vs pH 7.2, 7.4, 7.6, 7.8; pH 7.2 vs pH 7.4, 7.6, 7.8; pH 7.4 vs pH 7.6, 7.8 (*P* < 0.001); pH 7.6 vs pH 7.8 (*P* < 0.05). d = pH 6.8 vs pH 7.0, 7.2, 7.4, 7.6, 7.8; pH 7.0 vs pH 7.2, 7.4, 7.6, 7.8; pH 7.2 vs pH 7.4, 7.6, 7.8 (*P* < 0.001); pH 7.4 vs pH 7.6 (*P* < 0.05). e = pH 6.8 vs pH 7.0, 7.2, 7.4, 7.6, 7.8; pH 7.0 vs pH 7.2, 7.4, 7.6, 7.8; pH 7.2 vs pH 7.6, 7.8 (*P* < 0.001); pH 7.4 vs pH 7.2, 7.8 (*P* < 0.05). Time course interconversion of PVL shows disappearance of PVL (**C**) and formation of pravastatin hydroxy acid form (**D**). a = pH 7.6 vs pH 6.8, 7.4 (*P* < 0.01); pH 7.8 vs pH 6.8, 7.0, 7.2, 7.4; pH 7.2 vs pH 7.6 (*P* < 0.001). b = pH 6.8 vs pH 7.2, 7.4, 7.6, 7.8; pH 7.0 vs pH 7.2, 7.4, 7.6, 7.8; pH 7.2 vs pH 7.4, 7.6, 7.8; pH 7.4 vs pH 7.6, 7.8; pH 7.6 vs pH 7.8 (*P* < 0.001). c = pH 6.8 vs pH 7.0, 7.2, 7.4, 7.6, 7.8; pH 7.0 vs pH 7.2, 7.4, 7.6, 7.8; pH 7.2 vs pH 7.4, 7.6, 7.8; pH 7.4 vs pH 7.6, 7.8 (*P* < 0.001). d = pH 6.8 vs pH 7.0, 7.2, 7.4, 7.6, 7.8; pH 7.0 vs pH 7.4, 7.6, 7.8 (*P* < 0.001); pH 7.0 vs pH 7.2 (*P* < 0.01).

Because only the lactone form of statins underwent pH-dependent conversion, time course conversion studies were performed for only this form of simvastatin and pravastatin at a concentration of 50 μmol/L and pH of 6.8–7.8 over 48 hours in human plasma (Fig 3), PBS (Supplementary Fig 3), and DMEM culture medium (Supplementary Fig 4). It is clear that lactone hydrolysis is both pH- and time-dependent. Even a slight increase in pH is accompanied by substantial increase in the rate of hydrolysis to the less lipophilic acid form. This conversion was observed as early as 30 minutes (Fig 3, Supplementary Figs 3 and 4). After 48 hours of incubation with any matrix of alkaline pH, the lactone form of simvastatin and pravastatin was almost completely converted to the hydroxy acid form. However, at acidic pH, substantial parts of simvastatin and pravastatin from different matrices remain in the lactone form. The exception is PVL incubated with human plasma which shows almost complete conversion to the hydroxy acid form after 48 hours even under acidic condition. Faster rate of hydrolysis was observed with PVL incubated with human plasma compared to its incubation with DMEM and PBS. Contrary to this, the rate of hydrolysis of SVL was slower in plasma samples than in DMEM and PBS samples. This was demonstrated by comparing the hydrolysis half-life of the lactone form of statins under different pH levels (Table I). PVL showed the shortest half-life when incubated with human plasma, whereas SVL demonstrated the longest half-life. Identical interconversion patterns were reported for each statin over all tested concentrations (12.5, 25, 50, and 100 μmol/L for SVL and SVA and 25, 50, 100, and 200 μmol/L for PVL and PVA) and within different matrices (data not shown).

### Liver microsomal metabolic stability

**Fig 4:**
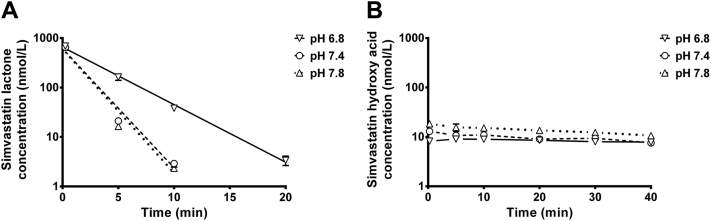
Liver microsomal stability of simvastatin lactone at 3 different pH levels. (**A**) Microsomal stability of simvastatin lactone as a function of time at different pH levels; (**B**) concentration-time profiles of simvastatin hydroxy acid form detected in liver microsomal stability reaction mixtures at 3 different pH levels. Results are expressed as mean ± standard deviation, (n = 3). Solid line denotes exponential regression of samples at pH 6.8; dashed line denotes exponential regression of samples at pH 7.4; whereas dotted line denotes exponential regression samples at pH 7.8.

Stability profiles of SVL at different pH levels and concentration-time profiles of SVA form in corresponding reaction mixtures are depicted in Fig 4, *A* and *B*, respectively. SVL was metabolized at slower rate in the microsomal reaction mixture at pH 6.8 compared with other pH conditions; the half-lives (mean ± SD) were 2.61 ± 0.07, 1.28 ± 0.05, and 1.23 ± 0.01 minutes at pH 6.8, 7.4, and 7.8, respectively. It is worth mentioning that the concentration-time profiles of SVA form seen in Fig 4, *B* do not correspond to the rate of metabolic loss of the lactone, indicating that the hydroxy acid is not the main metabolite of SVL in the reaction mixture.

### Characterization of myogenic differentiation of C2C12

**Fig 5:**
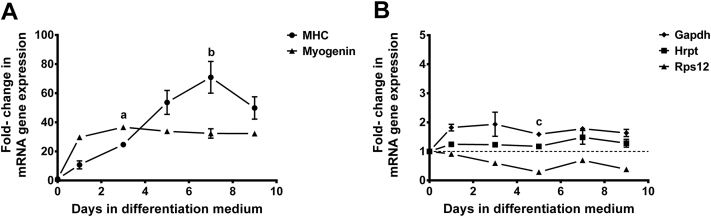
qPCR expression profiles of (**A**) myogenin and myosin heavy chain at days 0–9 of C2C12 differentiation; (**B**) glyceraldehyde-3-phosphate dehydrogenase, hypoxanthine-guanine phosphoribosyltransferase and ribosomal protein S12 reference genes at days 0–9 of C2C12 differentiation. Analysis of gene expression was done after normalization to single reference gene (TATA box-binding protein) and the fold changes in gene expression were expressed relative to gene expression in undifferentiated cells. Results are expressed as mean ± standard deviation, (n = 3). Data were analyzed by Kruskal-Wallis nonparametric test followed by Dunn's test for multiple comparisons. a = significant difference in myogenin expression between day 0 and day 3 (*P* < 0.01); b = significant difference in myosin heavy chain expression between day 0 and day 7 (*P* < 0.01); c = significant difference in ribosomal protein S12 expression between day 0 and day 5 (*P* < 0.05). mRNA, messenger RNA.

Analysis of real-time qPCR results revealed the presence of marked increase in the expression of myogenin and *MHC* genes during C2C12 differentiation (Fig 5, *A*). Myogenin expression was increased in the early stage of myoblast differentiation, reached a maximum of 36-fold the baseline levels after 3 days and remain elevated during the subsequent days of differentiation. On the other hand, a gradual increase in gene expression was observed with *MHC* gene, and the levels of expression reached a maximum of 70-fold by day 7. No significant changes were reported in the expression of *Gapdh*, *Hprt* and at most time points of *Rps12* reference genes during C2C12 cell differentiation (Fig 5, *B*).

### Cellular uptake of statins in response to altered medium pH

To gain a better understanding of the influence of pH alteration on statin-induced myotoxicity, time course uptake studies of SVL, SVA, PVL, and PVA were performed using undifferentiated and differentiated C2C12 cells over pH relevant to acidosis, alkalosis, and physiological conditions.

**Supplementary Fig 5:**
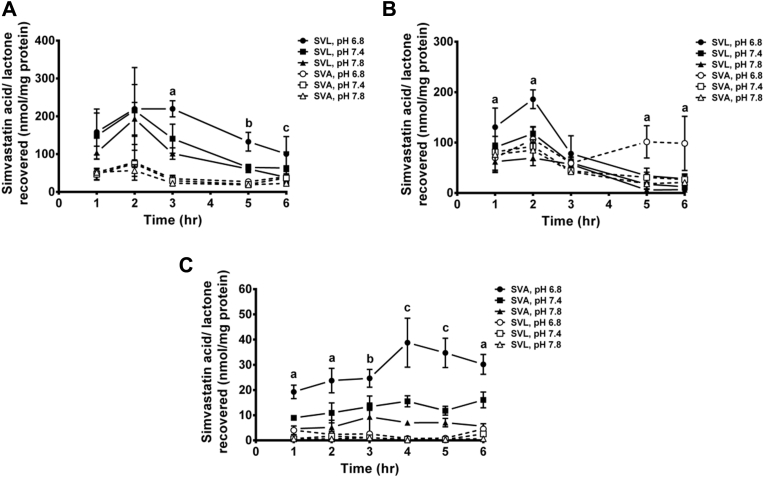
Uptake of simvastatin by undifferentiated and differentiated C2C12 cells. The uptake was determined after incubation of the cells with 1 μmol/L of simvastatin lactone (SVL) or simvastatin hydroxy acid (SVA) in Dulbecco's modified eagle medium of different pH levels (6.8–7.8) at 37°C for 6 hours. Results are expressed as nanomoles per milligram of protein ± standard deviation, (n = 5). Data were analyzed by 2-way analysis of variance followed by Bonferroni's post hoc test. (**A**) Intracellular concentration of simvastatin acid and lactone recovered from undifferentiated cells after treatment with 1 μmol/L SVL. a = SVL, pH 6.8 vs SVA, pH 6.8; SVL, pH 7.4 vs SVA, pH 7.4 (*P* < 0.001); b = SVL, pH 6.8 vs SVA, pH 6.8; SVL, pH 7.4 vs SVA, pH 7.4; SVL, pH 7.8 vs SVA, pH 7.8 (*P* < 0.001); c = SVL, pH 6.8 vs SVA, pH 6.8; SVL, pH 7.4 vs SVA, pH 7.4 (*P* < 0.001); SVL, pH 7.8 vs SVA, pH 7.8 (*P* < 0.01). (**B**) Intracellular concentration of simvastatin acid and lactone recovered from differentiated cells after treatment with 1 μmol/L SVL. a = SVL, pH 6.8 vs SVA, pH 6.8 (*P* < 0.001). (**C**) Intracellular concentration of simvastatin acid and lactone recovered from differentiated cells after treatment with 1 μmol/L SVA. a = SVA, pH 6.8 vs SVL, pH 6.8; SVA, pH 7.4 vs SVL, pH 7.4 (*P* < 0.001); b = SVA, pH 6.8 vs SVL, pH 6.8; SVA, pH 7.4 vs SVL, pH 7.4; SVA, pH 7.8 vs SVL, pH 7.8 (*P* < 0.001); c = SVA, pH 6.8 vs SVL, pH 6.8; SVA, pH 7.4 vs SVL, pH 7.4 (*P* < 0.001); SVA, pH 7.8 vs SVL, pH 7.8 (*P* < 0.01).

**Fig 6:**
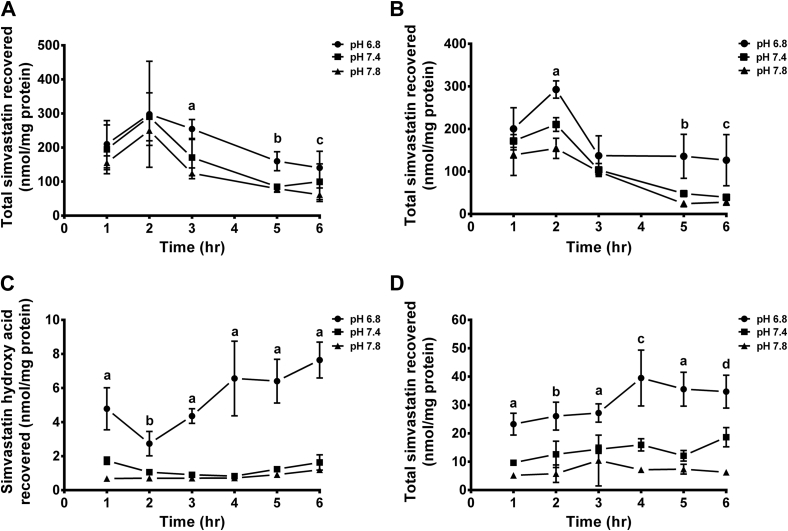
Uptake of simvastatin lactone (SVL) and simvastatin hydroxy acid (SVA) by undifferentiated and differentiated C2C12 cells. The uptake was determined after incubation of the cells with 1 μmol/L of SVL or SVA in Dulbecco's modified eagle medium culture medium of different pH levels (6.8–7.8) at 37°C for 6 hours. Results are expressed as nanomoles per milligram of protein ±standard deviation, (n = 5). Data were analyzed by 2-way analysis of variance followed by Bonferroni's post hoc test. (**A**) Total simvastatin (lactone + hydroxy acid) recovered by undifferentiated C2C12 cells after treatment with 1 μmol/L SVL. a = pH 6.8 vs pH 7.4 (*P* < 0.01); pH 6.8 vs pH 7.8 (*P* < 0.001); b = pH 6.8 vs pH 7.4, 7.8 (*P* < 0.001); c = pH 6.8 vs pH 7.8 (*P* < 0.05). (**B**) Total simvastatin recovered by differentiated C2C12 cells after treatment with 1 μmol/L SVL. a = pH 6.8 vs pH 7.4, 7.8 (*P* < 0.001); pH 7.4 vs pH 7.8 (*P* < 0.01); b = pH 6.8 vs pH 7.4 (*P* < 0.01); pH 6.8 vs pH 7.8 (*P* < 0.001); c = pH 6.8 vs pH 7.4, 7.8 (*P* < 0.01). (**C**) SVA recovered by undifferentiated C2C12 cells after treatment with 1 μmol/L SVA (no SVL was recovered in this experiment). a = pH 6.8 vs pH 7.4 (*P* < 0.01); pH 6.8 vs pH 7.8 (*P* < 0.001); b = pH 6.8 vs pH 7.4, 7.8 (*P* < 0.001). (**D**) Total simvastatin recovered by differentiated C2C12 cells after treatment with 1 μmol/L SVA. a = pH 6.8 vs pH 7.4, 7.8 (*P* < 0.001); b = pH 6.8 vs pH 7.4, 7.8 (*P* < 0.001); pH 7.4 vs pH 7.8 (*P* < 0.05); c = pH 6.8 vs pH 7.4, 7.8 (*P* < 0.001); pH 7.4 vs pH 7.8 (*P* < 0.01); d = pH 6.8 vs pH 7.4, 7.8; pH 7.4 vs pH 7.8 (*P* < 0.001).

The effect of medium pH alteration on cellular simvastatin uptake was assessed by comparing the concentration of simvastatin recovered from muscle cells maintained under different pH conditions. Fig 6 shows the uptake of simvastatin by undifferentiated and differentiated C2C12 after treatment with 1 μmol/L SVL or SVA over period of 6 hours. The maximum cellular uptake of SVL by undifferentiated cells was reached within 2 hours of treatment. This initial increase in cellular SVL uptake by muscle cells was followed by a gradual decline over time (Fig 6, *A*). During the first 2 hours of treatment, no significant differences in SVL uptake by undifferentiated cells were found between different pH levels. However, incubating cells for longer period of time resulted in significant differences in the cellular SVL uptake at different pH levels as shown by the changes in the concentration of total simvastatin (lactone and hydroxy acid forms) recovered in response to medium pH modifications (Fig 6, *A*). The total simvastatin recovered from undifferentiated cells maintained at physiological pH after 3, 5, and 6 hours of treatment was 170.5 ± 23.7, 84.8 ± 4.1, and 99.7 ± 21.4 nmol/mg protein, respectively. Reducing extracellular pH to acidic range resulted in a marked increase in total simvastatin uptake by approximately 50%, 78%, and 40%, respectively, compared with total simvastatin recovered from cells grown under physiological pH. On the other hand, no significant differences of SVL uptake were detected between cells grown under alkaline and physiological conditions (Fig 6, *A*). The intracellular concentrations of SVL recovered from undifferentiated C2C12 cells were significantly higher than those of the hydroxy acid form at all tested pH levels (Supplementary Fig 5, *A*).

Unlike undifferentiated cells, short-term treatment of differentiated C2C12 cells with SVL for 2-hour period resulted in significant changes in simvastatin uptake in response to medium pH alteration. At physiological pH, after 2 hours of treatment, SVL uptake by differentiated C2C12 cells was 210.4 ± 7.2 nmol/mg protein. Acidification of culture medium to a pH of 6.8 significantly increased SVL uptake by 40% relative to physiological pH, whereas medium alkalinization to a pH of 7.8 resulted in a significant reduction in SVL uptake by 27% (Fig 6, *B*).

Results of cellular uptake of SVA by undifferentiated and differentiated C2C12 cells under different pH conditions are shown in Fig 6, *C* and *D*, respectively. The uptake of SVA by these cells was much lower than that of SVL at all time points. Significantly more SVA (∼2.5–5 folds) was recovered form undifferentiated C2C12 cells maintained under acidic pH compared with cells treated under physiological conditions. Increasing the extracellular pH to alkaline level of 7.8 has not been shown to further increase the uptake of SVA compared to pH 7.4 (Fig 6, *C*). All intracellular simvastatin recovered from undifferentiated cells was in the hydroxy acid form, and no SVL was detected at any pH level.

The uptake of SVA by differentiated C2C12 was several folds higher than their uptake by undifferentiated cells. Reducing the extracellular pH to acidic levels significantly increased the uptake of SVA by differentiated C2C12 cells by more than 2 folds, whereas maintaining the cells under alkaline pH significantly reduced their uptake by approximately 50% (Fig 6, *D*). Unlike undifferentiated cells, it was possible to observe minor interconversion of SVA to the corresponding lactone form by differentiated cells treated under different pH levels (Supplementary Fig 5, *C*).

Uptake study was also performed by incubating undifferentiated and differentiated C2C12 cells with PVL or PVA (1 μmol/L). As expected, the intracellular levels of pravastatin were extremely low (below the limit of quantification of the analytical assay; data not shown).

### Expression of cellular transporters

**Supplementary Fig 10:**
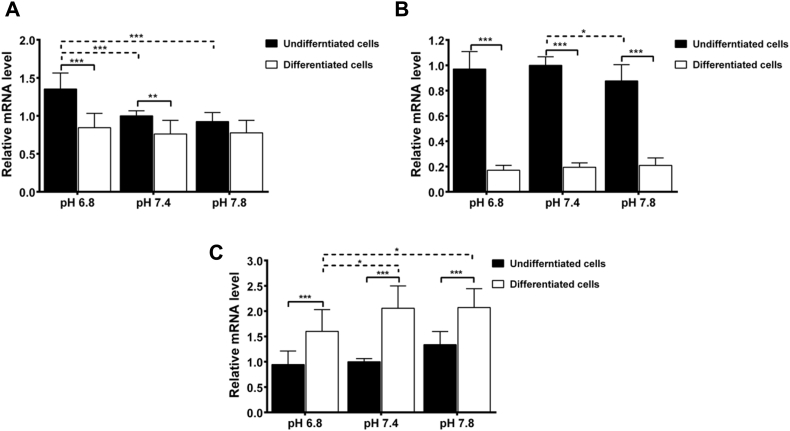
Messenger RNA expression of (**A**) *MRP1*, (**B**) *MRP4*, and (**C**) *MRP5* in undifferentiated and differentiated C2C12 cells maintained at different pH levels. Results are expressed as fold changes in gene expression relative to the baseline level observed with undifferentiated C2C12 maintained at physiological pH and were normalized relative to *Gapdh*. Data are presented as mean ± standard deviation, (n = 4) and were analyzed by 2-way analysis of variance followed by Bonferroni's post hoc test, ^∗∗∗^*P* < 0.001, ^∗∗^*P* < 0.01, ^∗^*P* < 0.05. Solid lines denote differences in relative messenger RNA levels between undifferentiated and differentiated cells, whereas dashed lines denote differences within each cell type maintained at different pH levels.

**Supplementary Fig 11:**
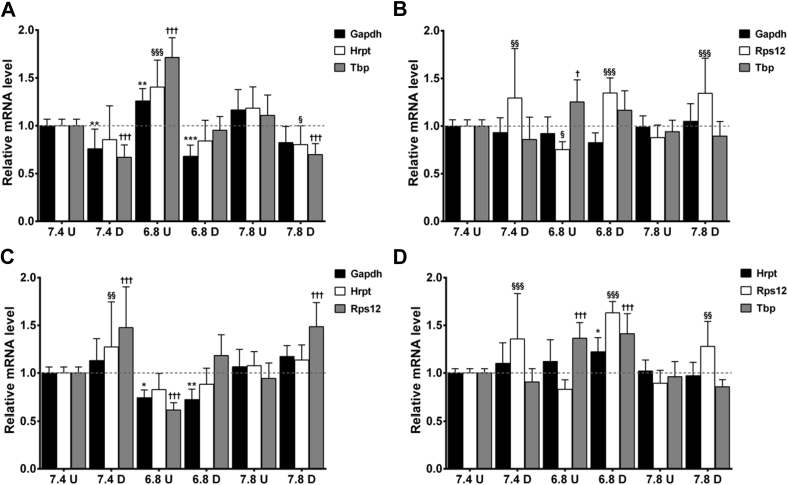
Messenger RNA expression of (**A**) ribosomal protein S12, (**B**) hypoxanthine phosphoribosyltransferase, (**C**) TATA box-binding protein, and (**D**) glyceraldehyde-3-phosphate dehydrogenase reference genes. Results are expressed as fold changes in gene expression relative to the baseline level observed with undifferentiated C2C12 maintained at physiological pH. Each reference gene was normalized to the other 3 reference genes. Data are presented as mean ± standard deviation, (n = 4) and were analyzed 2-way analysis of variance followed by Bonferroni's post hoc test, ^∗∗∗, §§§, †††^*P* < 0.001, ^∗∗, §§^*P* < 0.01, ^∗, §, †^*P* < 0.05 compared with messenger RNA expression level in undifferentiated cell samples of pH 7.4.

In the present study, the expression of mRNA of 2 influx transporters (*OATP1a4* and *OATP2b1*) and 3 efflux transporters (*MRP1*, *MRP4*, and *MRP5*) were assessed in undifferentiated and differentiated C2C12 cells maintained under different pH conditions. These transporters have been reported to be involved in statin uptake.^46^, ^47^, ^48^ As shown in Supplementary Fig 10, *MRP1*, *MRP4*, and *MRP5* were expressed in both cell types. However, neither *OATP1a4* nor *OATP2b1* influx transporters were detected in either cell type. This is consistent with previous reports that showed no expression of these influx transporters in undifferentiated and differentiated C2C12 cells lines.^46^, ^48^ The mRNA of both *MRP1* and *MRP4* efflux transporters were found to be expressed at higher levels in undifferentiated cells, whereas *MRP5* showed higher expression in differentiated cells. The pH dependency was found with *MRP1* in undifferentiated cells and with *MRP5* in differentiated cells. *MRP1* showed higher expression at acidic pH in undifferentiated cells, whereas *MRP5* exhibited increased levels with alkaline pH in differentiated cells. Results of mRNA expression of reference genes are presented in Supplementary Fig 11.

### In vitro cytotoxicity of statins in different medium pH levels

**Fig 7:**
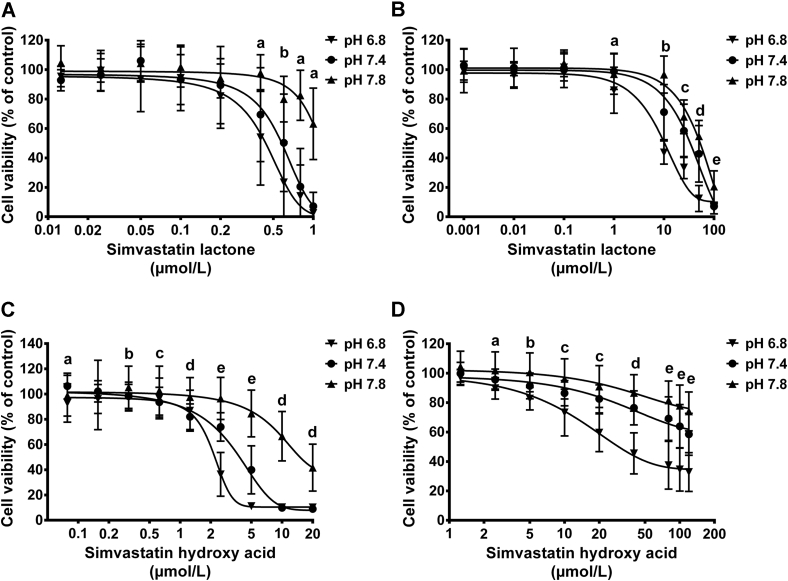
Effect of simvastatin lactone (SVL) and simvastatin hydroxy acid (SVA) on the viability of undifferentiated and differentiated C2C12. Cells were cultured at a density of 4,000 cells/well and allowed to attach for 24 hours or to differentiate for 4 days, then exposed to increasing concentrations of SVL or SVA under acidic, neutral, and alkaline medium pH for 72 hours. Results are presented as mean ± standard deviation of 3 experiments, 8 replicates per experiment. Data were analyzed by 2-way analysis of variance followed by Bonferroni's post hoc test. (**A**) Effects of SVL on cell viability of undifferentiated C2C12 myoblasts. a = pH 7.8 vs pH 6.8, 7.4 (*P* < 0.001); b = pH 7.8 vs pH 6.8, 7.4; pH 6.8 vs pH 7.4 (*P* < 0.001). (**B**) Effects of SVL on cell viability of differentiated C2C12 myocytes. a = pH 6.8 vs pH 7.4 (*P* < 0.01); pH 6.8 vs pH 7.8 (*P* < 0.05); b = pH 6.8 vs pH 7.4, 7.8; pH 7.4 vs pH 7.8 (*P* < 0.001); c = pH 6.8 vs pH 7.4, 7.8 (*P* < 0.001); d = pH 6.8 vs pH 7.4, 7.8 (*P* < 0.001); pH 7.4 vs pH 7.8 (*P* < 0.01); e = pH 7.8 vs pH 6.8, 7.4 (*P* < 0.001). (**C**) Effects of SVA on cell viability of undifferentiated C2C12 myoblasts. a = pH 6.8 vs pH 7.4 (*P* < 0.001); pH 7.4 vs pH 7.8 (*P* < 0.05); b = pH 7.8 vs pH 6.8, 7.4 (*P* < 0.05); c = pH 7.4 vs pH 7.8 (*P* < 0.01); d = pH 7.8 vs pH 6.8, 7.4 (*P* < 0.001); e = pH 6.8 vs pH 7.4, 7.8; pH 7.4 vs pH 7.8 (*P* < 0.001). (**D**) Effects of SVA on cell viability of differentiated C2C12 myocytes. a = pH 6.8 vs pH 7.8 (*P* < 0.001); b = pH 7.4 vs pH 6.8, 7.8 (*P* < 0.05); pH 6.8 vs pH 7.8 (*P* < 0.001); c = pH 6.8 vs pH 7.4, 7.8 (*P* < 0.001); pH 7.4 vs pH 7.8 (*P* < 0.05); d = pH 6.8 vs pH 7.4, 7.8 (*P* < 0.001); pH 7.4 vs pH 7.8 (*P* < 0.01); e = pH 6.8 vs pH 7.4, 7.8; pH 7.4 vs pH 7.8 (*P* < 0.001).

**Fig 8:**
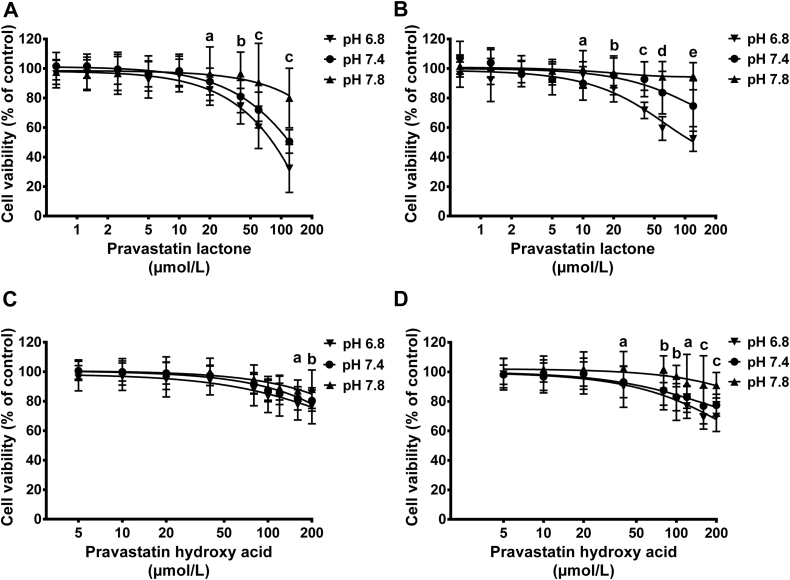
Effect of pravastatin lactone (PVL) and pravastatin hydroxy acid (PVA) on the viability of undifferentiated and differentiated C2C12. Cells were cultured at a density of 4,000 cells/well and allowed to attach for 24 hours or to differentiate for 4 days, then exposed to increasing concentrations of PVL or PVA under acidic, neutral, and alkaline medium pH for 72 hours. Results are presented as mean ± standard deviation of 3 experiments, 8 replicates per experiment. Data were analyzed by 2-way analysis of variance followed by Bonferroni's post hoc test. (**A**) Effects of PVL on cell viability of undifferentiated C2C12 myoblasts. a = pH 6.8 vs pH 7.8 (*P* < 0.001); b = pH 6.8 vs pH 7.4 (*P* < 0.05); pH 7.8 vs pH 6.8, 7.4 (*P* < 0.001); c = pH 6.8 vs pH 7.4, 7.8, pH 7.4 vs pH 7.8 (*P* < 0.001). (**B**) Effects of PVL on cell viability of differentiated C2C12 myocytes. a = pH 7.4 vs pH 7.8 (*P* < 0.05); b = pH 6.8 vs pH 7.8 (*P* < 0.01); c = pH 6.8 vs pH 7.4, 7.8 (*P* < 0.001); d = pH 6.8 vs pH 7.4, 7.8 (*P* < 0.001), pH 7.4 vs pH 7.8 (*P* < 0.01); e = pH 6.8 vs pH 7.4, 7.8, pH 7.4 vs pH 7.8 (*P* < 0.001). (**C**) Effect of PVA on cell viability of undifferentiated C2C12 myoblasts. a = pH 6.8 vs pH 7.8 (*P* < 0.01); b = pH 6.8 vs pH 7.8 (*P* < 0.001). (**D**) Effect of PVA on cell viability of differentiated C2C12 myocytes. a = pH 6.8 vs pH 7.8 (*P* < 0.001); pH 7.4 vs pH 7.8 (*P* < 0.01); b = pH 7.8 vs pH 6.8, 7.4 (*P* < 0.001); c = pH 7.8 vs pH 6.8, 7.4 (*P* < 0.001); pH 6.8 vs pH 7.4 (*P* < 0.05).

The effect of medium pH changes on cytotoxicity of statins was investigated using both undifferentiated and differentiated C2C12 skeletal muscle cells. Cells were exposed to increasing concentrations of SVL, SVA, PVL, or PVA for 72 hours, and the metabolic activity of the cells was measured using the MTT test (Figs 7 and 8). The IC_50_ of SVL in undifferentiated C2C12 cells grown in DMEM culture medium of physiological pH was found to be 0.58 ± 0.02 μmol/L. Medium acidification to a pH of 6.8 significantly reduced the IC_50_ to 0.44 ± 0.02 μmol/L, whereas alkalinizing the medium to pH 7.8 resulted in significant increase in IC_50_ to 1.13 ± 0.04 μmol/L. Similar cytotoxicity pattern was observed with differentiated C2C12 cells treated with SVL under different pH conditions (Table II). Likewise, incubating C2C12 cells with SVA resulted in cytotoxic response comparable with that observed with SVL in response to medium pH alteration (Fig 7). However, the IC_50_ values of SVA were several folds higher than that of SVL (Table II). The higher cytotoxicity of SVA observed at acidic pH was associated with enhanced cellular uptake. The cytotoxicity of PVL and PVA in both undifferentiated and differentiated C2C12 was much lower than that of SVL and SVA after 72 hours of treatment under different pH conditions (Figs 7 and 8). Interestingly, undifferentiated C2C12 cells were found to be more sensitive to statin-induced myotoxicity compared with differentiated cells (Table II).

**Fig 9:**
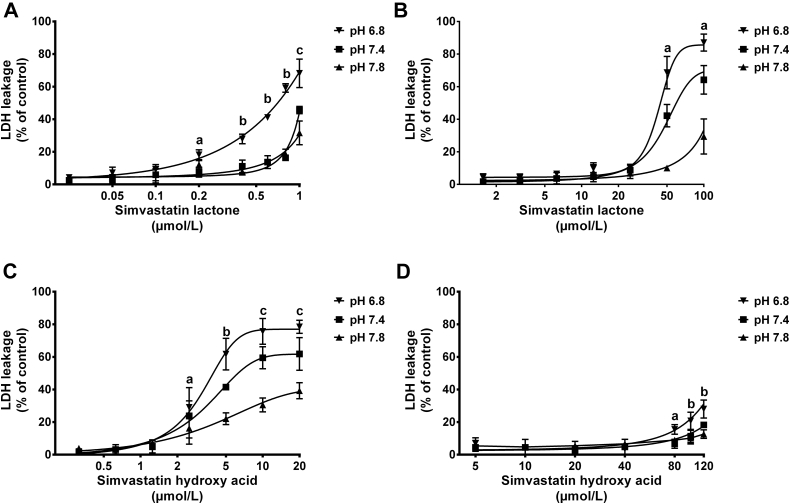
Effect of simvastatin lactone (SVL) and simvastatin hydroxy acid (SVA) on lactate dehydrogenase (LDH) release from undifferentiated and differentiated C2C12 cells maintained under different pH levels. C2C12 cells were cultured at a density of 4,000 cells/well and allowed to attach for 24 hours or to differentiate for 4 days, then exposed to increasing concentrations of SVL or SVA under acidic, neutral, and alkaline medium pH. Undifferentiated cells were treated for 72 hours, whereas differentiated cells were maintained for 24 hours. Data are presented as mean ± standard deviation, (n = 3) and analyzed by 2-way analysis of variance followed by Bonferroni's post hoc test. (**A**) LDH release from undifferentiated C2C12 cells treated with SVL. a = pH 6.8 vs pH 7.4 (*P* < 0.001); b = pH 6.8 vs pH 7.4, 7.8 (*P* < 0.001); c = pH 6.8 vs pH 7.4, 7.8; pH 7.4 vs pH 7.8 (*P* < 0.001). (**B**) LDH release from differentiated C2C12 cells treated with SVL. a = pH 6.8 vs pH 7.4, 7.8; pH 7.4 vs pH 7.8 (*P* < 0.001). (**C**) LDH release from undifferentiated C2C12 cells treated with SVA. a = pH 6.8 vs pH 7.8 (*P* < 0.05); b = pH 6.8 vs pH 7.4, 7.8; pH 7.4 vs pH 7.8 (*P* < 0.001); c = pH 6.8 vs pH 7.4 (*P* < 0.01); pH 7.8 vs pH 6.8, 7.4 (*P* < 0.001). (**D**) LDH release from differentiated C2C12 cells treated with SVA. a = pH 6.8 vs pH 7.4 (*P* < 0.01); pH 6.8 vs pH 7.8 (*P* < 0.05); b = pH 6.8 vs pH 7.4, 7.8 (*P* < 0.0001).

**Fig 10:**
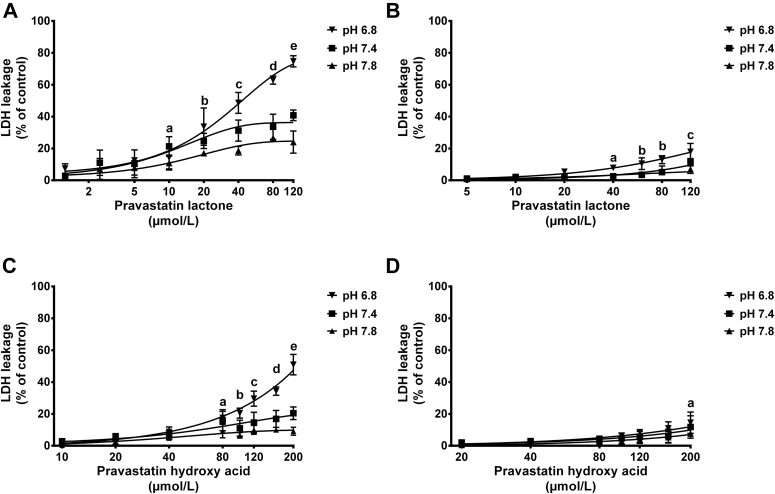
Effect of pravastatin lactone (PVL) and pravastatin hydroxy acid (PVA) on lactate dehydrogenase (LDH) release from undifferentiated and differentiated C2C12 cells maintained under different pH levels. C2C12 cells were cultured at a density of 4,000 cells/well and allowed to attach for 24 hours or to differentiate for 4 days, then exposed to increasing concentrations of PVL or PVA under acidic, neutral, and alkaline medium pH. Undifferentiated cells were treated for 72 hours, whereas differentiated cells were maintained for 24 hours. Data are presented as mean ± standard deviation, (n = 3) and analyzed by 2-way analysis of variance followed by Bonferroni's post hoc test. (**A**) LDH release from undifferentiated C2C12 cells treated with PVL. a = pH 7.4 vs pH 7.8 (*P* < 0.05); b = pH 6.8 vs pH 7.8 (*P* < 0.001); c = pH 6.8 vs pH 7.4, 7.8 (*P* < 0.001); pH 7.4 vs pH 7.8 (*P* < 0.05); d = pH 6.8 vs pH 7.4, 7.8 (*P* < 0.001); e = pH 6.8 vs pH 7.4, 7.8; pH 7.4 vs pH 7.8 (*P* < 0.001). (**B**) LDH release from differentiated C2C12 cells treated with PVL. a = pH 6.8 vs pH 7.4, 7.8 (*P* < 0.01); b = pH 6.8 vs pH 7.4, 7.8 (*P* < 0.001); c = pH 6.8 vs pH 7.4, 7.8 (*P* < 0.001); pH 7.4 vs pH 7.8 (*P* < 0.05). (**C**) LDH release from undifferentiated C2C12 cells treated with PVA. a = pH 6.8 vs pH 7.8 (*P* < 0.05); b = pH 6.8 vs pH 7.4 (*P* < 0.01); pH 6.8 vs pH 7.8 (*P* < 0.001); c = pH 6.8 vs pH 7.4, 7.8 (*P* < 0.001); d = pH 6.8 vs pH 7.4, 7.8 (*P* < 0.001); pH 7.4 vs pH 7.8 (*P* < 0.05); e = pH 6.8 vs pH 7.4, 7.8; pH 7.4 vs pH 7.8 (*P* < 0.001). (**D**) LDH release from differentiated C2C12 cells treated with PVA. a = pH 6.8 vs pH 7.8 *(P* < 0.05).

The effect of medium pH alteration on cell membrane integrity was also tested using LDH assay. Results from LDH release assay are shown in Figs 9 and 10. It is clear that simvastatin (applied either as lactone or hydroxy acid form) induces higher LDH leakage from cell membranes of undifferentiated and differentiated C2C12 cells than pravastatin over the tested concentrations. Compared with their hydroxy acid counterparts, the highly lipophilic lactone forms of simvastatin and pravastatin were shown to induce higher LDH release especially when cells were maintained under acidic conditions. The cytotoxicity results obtained with LDH assay correspond well with the cell viability data observed with MTT assay.

## Discussion

### Interconversion of statins in human plasma, PBS, and DMEM culture medium

Simvastatin has been selected in this work as a model lipophilic statin because of its wide clinical use, high lipophilicity, and high incidence of muscle toxicity.^2^, ^33^ Pravastatin was selected as a model hydrophilic statin with expected lower cellular uptake and myotoxicity. The interconversion of statins between lactone and hydroxy acid forms was investigated over concentrations ranging from 12.5 to 100 μmol/L for SVL and SVA and concentrations of 25 to 200 μmol/L for PVL and PVA. These concentrations have been selected based on a review of literature that described the in vitro experiments of statin-induced myotoxicity.^49^, ^50^, ^51^, ^52^, ^53^, ^54^

In the present study, the lactone forms of simvastatin and pravastatin were found to be highly susceptible to pH-dependent conversion to the less lipophilic hydroxy acid forms. Acidification of human plasma, PBS, and DMEM culture medium significantly reduced the conversion of the lactone forms of both statins to the corresponding acid forms. On the other hand, alkalinization resulted in almost complete conversion of the lactone form to the active and less myotoxic hydroxy acid form (Fig 2, panel A and C). It is worth noting that regardless of the different pH conditions, the hydroxy acid forms of simvastatin and pravastatin were more stable than the lactone forms (Fig 2, panel B and D).

Statins are known to have relatively short half-lives in the range of 1–5 hours.^55^ However, because the effects of pH alteration on statin interconversion were observed within the first 30 minutes of incubation (Fig 3, Supplementary Figs 3 and 4), changes in plasma pH are expected to affect statins interconversion within the general circulation before statin being cleared from the body. A slower rate of hydrolysis was reported when SVL was incubated with human plasma compared with DMEM and PBS samples as shown by longer half-life observed under acidic conditions (Table I). This finding could be attributed to the binding of SVL to albumin and other plasma proteins. It has been reported that SVL is extensively bound to plasma proteins (95%–98%).^55^ Such binding reduces the free (unbound) fraction of SVL, makes it less susceptible to pH-dependent interconversion, and consequently slows down its hydrolysis. Similar results were reported with camptothecin (an anticancer drug which undergoes a similar pH-dependent interconversion between lactone and hydroxy acid forms).^56^, ^57^ On the other hand, the rate of hydrolysis of PVL from plasma samples was found to be faster than that from DMEM and PBS samples probably because of low plasma protein binding (50%) that render PVL more available for chemical and enzymatic hydrolysis (by plasma estreases and paraxonases).^58^, ^59^

### Liver microsomal metabolic stability

Metabolic stability studies were performed using human liver microsomes and potassium phosphate buffer at different pH levels to simulate acidosis, physiological pH, and alkalosis. SVL was tested in microsomal stability experiments because it was found to be the most cytotoxic statin in our studies. SVL underwent extensive metabolism at all pH levels, but at acidic pH, the metabolic rate was 2-fold slower than that at physiological pH. This result suggests that at pathophysiological conditions of acidosis, the metabolism of the lactone form by liver enzymes could be slower. Therefore, both the slower metabolism and slower chemical conversion rate of the lactone form would lead to higher levels of the lactone in general circulation. Interestingly, Fig 4, *B* shows that the levels of formation of SVA do not correspond to metabolic loss of the lactone form, which suggests that the hydroxy acid form is not the main metabolite formed in the microsomal reaction mixture. It is also evident that SVA is not metabolized extensively by the liver microsomes, which is in agreement with previously reported studies.^60^

### Characterization of myogenic differentiation of C2C12

In this study, successful differentiation of C2C12 myoblast cells into functioning and integrated myotubes was confirmed by examining the expression of 2 markers of myogenic differentiation (myogenin and *MHC*). The expression of myogenin started at day 1 after induction of differentiation and reached high levels at day 3, a stage at which the cells start fusing and form multinucleated myotubes. It has been suggested that the expression of myogenin (an early marker for the entry of C2C12 myoblasts into the differentiation phase) is followed by skeletal myogenesis through a highly organized sequence of events that involve withdrawal from cell cycle, expression of contractile proteins such as myosin heavy chain, and finally cell fusion resulting in the formation of multinucleated myotubes.^61^

On the other hand, the expression of *MHC* showed a steady increase over time and reached a maximum of 70-fold the baseline level by the seventh day. In previously reported study, western blot analysis of a number of myogenic markers during C2C12 differentiation revealed that the expression of myogenin preceded the induction of *MHC*, whose expression showed a steady increase up to 4 days in the differentiation medium.^61^

### Cellular uptake of statins in response to altered medium pH

High-plasma statin levels have been considered to be a risk factor for statin-induced myotoxicity. However, there are patients who exhibit high statin plasma levels but do not develop myopathy, suggesting that other factors, including skeletal muscle fiber statin concentration, may have an impact on side effect risk.^48^ In the present study, the alteration of extracellular pH as a result of acidosis and alkalosis has been proposed to contribute to variable uptake of statins by skeletal muscle cells.

Although no pravastatin lactone or hydroxy acid forms were detected in either undifferentiated and differentiated C2C12 cells at any pH levels after different incubation times, the cellular uptake of simvastatin was found to be strongly affected by the pH level of the incubation medium. Incubating undifferentiated C2C12 cells with SVL for a period exceeding 2 hours resulted in significantly higher total simvastatin accumulation within the cells under acidic condition compared with neutral and alkaline conditions (Fig 6, *A*). These findings could be attributed to the greater proportion of simvastatin that remains in the more lipophilic lactone form at acidic pH. The lactone form has higher ability to cross the cell membranes of muscle cells and achieve high intracellular concentrations. This could explain the higher cytotoxicity observed under acidic condition compared with alkaline and neutral conditions (Table II). It should be noted that the increase in simvastatin uptake under acidic conditions is also expected to result in enhanced lipid-lowering activity.

In contrast to acidic conditions, medium alkalinization did not influence the cellular uptake of simvastatin significantly in comparison to the uptake at physiological pH. SVL is less stable at alkaline pH and therefore dynamic equilibrium favors hydrolysis of the lactone ring to yield the hydrophilic hydroxy acid form. Higher intracellular concentrations of the lactone than its corresponding acid form were recovered from undifferentiated cells at all pH levels indicating that cellular accumulation of simvastatin is associated with its lipophilic nature (Supplementary Fig 5, *A*). A marked difference in lipophilicity exists between lactone and hydroxy acid forms of statins, with the lactone form being more lipophilic than the corresponding acid form.^9^, ^11^ The lipid-enriched membranes of muscle cells act as a barrier to hydrophilic statins, whereas it allow passive diffusion of lipophilic ones.^62^ It has been suggested that intracellular statin concentrations are also controlled by the dynamic interplay between uptake and efflux transporter activities.^48^ However, it is not yet clear whether these transporters have differential selectivity to the acid or lactone forms of statins. It is worth noting that the intracellular concentrations of the acid form of simvastatin recovered from undifferentiated cells were not significantly different at various pH levels studied, suggesting that intracellular microenvironment was not influenced by the extracellular pH changes (Supplementary Fig 5, *A*).

Similarly, medium acidification increased simvastatin uptake by differentiated C2C12 cells as a result of greater proportion of the highly lipophilic lactone form preserved under acidic conditions (Fig 6, *B*). Less SVL was taken up by differentiated C2C12 cells compared with undifferentiated cells. Although the reasons for this difference are not entirely clear, the denser organization of cells as they differentiate could contribute to lower uptake of the statins by differentiated C2C12 cells. More studies will be needed to clarify this mechanism.

Measurement of the intracellular concentrations of both forms of simvastatin revealed a faster rate of hydrolysis of the lactone form within differentiated C2C12 cells compared to undifferentiated cells (Supplementary Fig 5, *A* and *B*).The higher metabolic rate of differentiated cells could explain the faster rate of SVL hydrolysis.

SVA has been shown to be taken up by undifferentiated and differentiated C2C12 cells to much lower extent than SVL (Fig 6, *C* and *D*). The higher uptake of SVA observed under acidic conditions could be attributed to the passive diffusion of the more lipophilic unionized form of SVA which becomes more predominant at acidic pH compared with physiological and alkaline pH.

### Expression of cellular transporters

Potential contribution of drug transporters to cellular uptake and cytotoxicity of statins over different pH levels was assessed in this study by measuring the mRNA expression of influx and efflux transporters that are known to influence statin uptake. A recent study of statin myotoxicity has shown that differentiated human skeletal muscle cells constitutively express *MRP* transporters (namely *MRP1*, *MRP4*, and *MRP5*) which mediate the efflux of statins from skeletal muscle fibers. Expression of these efflux transporters combined with the inability of differentiated muscle cells grown in vitro to express OATPs influx transporters could potentially play protective roles against intracellular statin accumulation.^48^ Similar to previously reported studies,^46^, ^48^ the levels of mRNA expression of influx transporters (*OATP1a4* and *OATP2b1*) were not detectable in our experiments. *MRP5* was found to have higher expression in differentiated cells compared to undifferentiated cells which could explain the relative resistance of former cells to statin-induced cytotoxicity. Moreover, it was found to be expressed at a lower level at pH 6.8, where higher cytotoxicity was observed (Supplementary Fig 10). On the other hand, the mRNA expression of *MRP1* and *MRP4* transporter genes appear to be greater in undifferentiated compared with differentiated C2C12 cells.

Taken together, the results suggest that although *MRP5* could play some role in the intracellular accumulation of simvastatin, overall, the results of expression of transporters do not explain the intracellular accumulation of statins. Therefore, it is likely that passive diffusion of the more lipophilic lactone form is a primary mechanism of intracellular accumulation of statins, especially in acidic conditions.

### In vitro cytotoxicity of statins in different medium pH levels

#### MTT assay

For the cells to have a normal function and metabolism, the pH should be maintained within narrow limits of 7.35–7.45. Disturbances in acid-base balance impose profound effects on many aspects of drug action.^63^ Our results demonstrated that alteration in culture medium pH greatly influenced the cytotoxicity of statins (Figs 7 and 8). The higher cytotoxicity of SVL found in C2C12 cells treated under acidic conditions could be attributed to the higher proportion of statin that remained in the more lipophilic lactone form. Low cytotoxicity under alkaline condition could be due to inability of statins to achieve adequate intracellular concentrations as a result of conversion to the hydrophilic acid form. It has been reported that SVL is about 3 times more lipophilic than its corresponding hydroxy acid form^9^ and is, therefore, expected to achieve higher intracellular concentrations. Enhanced cytotoxicity of SVA has also been demonstrated at acidic pH, which could be due to the passive transport mechanisms of the predominantly unionized hydroxy acid form in acidic environment.^64^ Hydrophilic pravastatin was shown to be less cytotoxic than lipophilic simvastatin. Undifferentiated C2C12 cells were found to be more sensitive to simvastatin-induced myotoxicity compared with differentiated cells (Table II). These findings suggest that multinuclear skeletal muscle cells are more resistant to statin-induced myotoxicity compared with mononuclear stem cells. Although SVL is inactive with respect to lipid-lowering effect, it may still mediate muscular side effects, either through a direct toxic effect or through intracellular conversion to the hydroxy methyl glutaryl-Co enzyme A reductase–inhibiting acid form.^12^

#### LDH cytotoxicity assay

In the present study, LDH has been used to reflect the cell membrane integrity in response to cell exposure to statin therapy. The amounts of LDH release induced by statins treatment over different pH levels are shown in Figs 9 and 10. The cytotoxicity detected with LDH assay was in agreement with the cell viability profiles obtained with MTT assay. Similar LDH release pattern was observed with both simvastatin and pravastatin in response to medium pH alteration. A significant increase in LDH release was observed when cells were exposed to statins under acidic conditions compared with physiological and alkaline conditions. The LDH release was higher from C2C12 cells treated with simvastatin compared with pravastatin treated cells. Furthermore, the lactone forms of both statins have induced higher LDH release than the corresponding acid form. The higher release of LDH by statins under acidic conditions is expected to be due to the higher cellular uptake of the more lipophilic lactone form.

## Conclusions

This work demonstrates that acid-base imbalance affects the interconversion of statins between the lactone and hydroxy acid forms. The conversion of lactone form of statins to the corresponding hydroxy acid form is strongly pH dependent. At physiological and alkaline pH, the lactone form undergoes substantial conversion while this conversion occurs at lower extent under acidic conditions. On the contrary, the conversion of the hydroxy acid form to the corresponding lactone form is negligible at any pH level. Our data also demonstrate that lipophilic SVL is more cytotoxic to undifferentiated and differentiated skeletal muscle cells than more hydrophilic SVA, PVL, and PVA. Furthermore, undifferentiated C2C12 cells are more sensitive to statin-induced myotoxicity than differentiated muscle cells. Physiological pH and alkalosis can protect against statin-induced myotoxicity, whereas acidosis enhances statin-induced myotoxicity as demonstrated by increased cellular uptake of statins under acidic conditions. These findings provide novel insight into the mechanisms of statin-induced myotoxicity in the presence of acidosis and alkalosis. By minimizing the chemical and enzymatic hydrolysis of the lactone form, acidosis can enhance statins uptake by the skeletal muscle cells and consequently potentiate their myotoxicity. On the other hand, alkalosis can potentiate the hydrolysis of the lactone ring rendering statins less lipophilic and therefore limits their penetration into the skeletal muscles and increases their uptake by the liver. Our findings suggest more selective and conservative approach, as well as tighter monitoring of statin-related skeletal muscle symptoms when prescribing lipophilic statins to patients who are at risk of developing acidosis.

## Figures and Tables

**Supplementary Fig 6:**
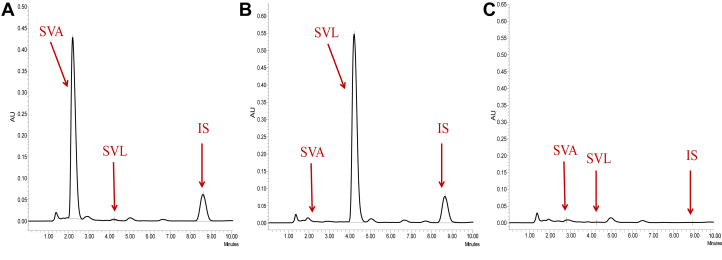
HPLC chromatograms of (**A**) SVA spiked into Dulbecco's modified eagle medium (pH 7.4) with internal standard (IS = 4,4-dichlorodiphenyltrichloroethane), (**B**) SVL spiked into Dulbecco's modified eagle medium (pH 7.4) with internal standard (IS = 4,4-dichlorodiphenyltrichloroethane), (**C**) blank culture medium. SVA, simvastatin hydroxy acid; SVL, simvastatin lactone; IS, internal standard.

**Supplementary Fig 7:**
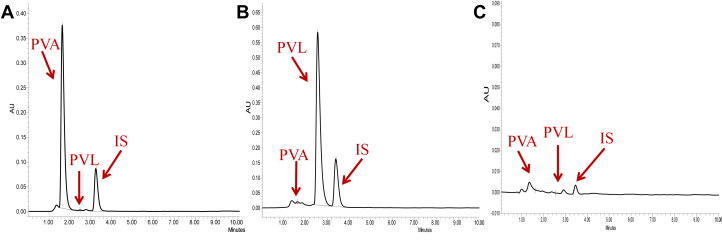
HPLC chromatograms of (**A**) PVA spiked into Dulbecco's modified eagle medium (pH 7.4) with internal standard (IS = griseofulvin), (**B**) PVL spiked into Dulbecco's modified eagle medium (pH 7.4) with internal standard (IS = griseofulvin), (**C**) blank culture medium. PVA, pravastatin hydroxy acid; PVL, pravastatin lactone; IS, internal standard.

**Supplementary Fig 8:**
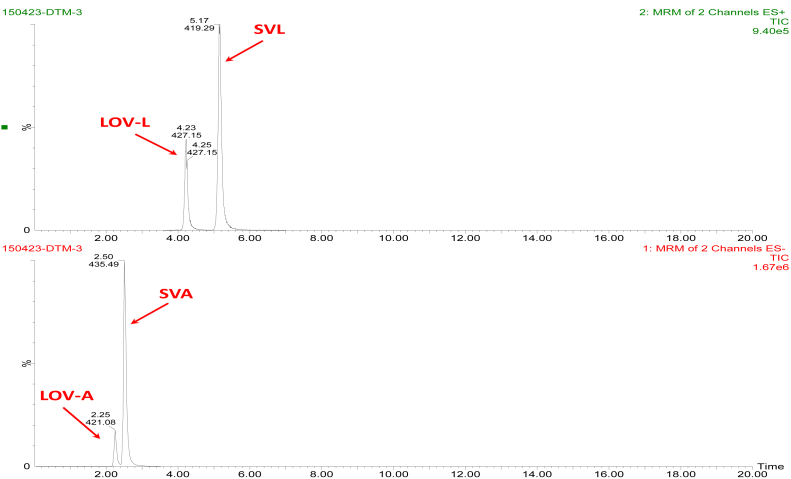
LC-MS/MS chromatograms of a mixture of measured analytes from cell lysate samples. LOV-A, lovastatin hydroxy acid (IS); SVA, simvastatin hydroxy acid; LOV-L, lovastatin lactone (IS); SVL, simvastatin lactone; IS, internal standard.

**Supplementary Fig 9:**
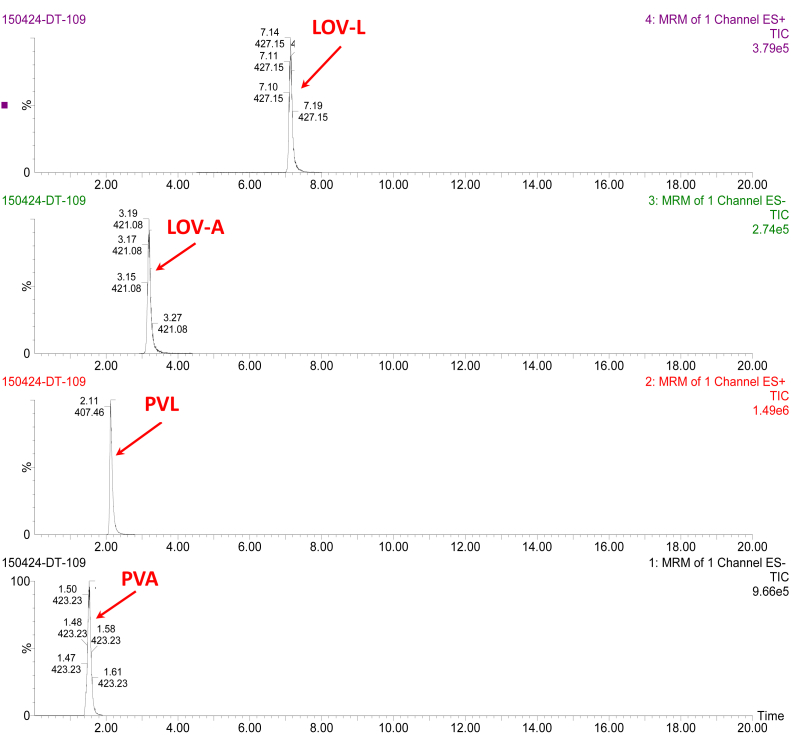
LC MS/MS chromatograms of a mixture of measured analytes from cell lysate samples. PVA, pravastatin hydroxy acid; PVL, pravastatin lactone; LOV-A, lovastatin hydroxy acid (IS); LOV-L, lovastatin lactone (IS); IS, internal standard.

**Supplementary Table I:** Primers used for gene expression quantification of differentiation markers

Gene	Direction	Primer sequence (5′-3′)	Melting temperature (°C)	Amplicon length (bp)∗
Myogenin	Forward	CAGGAGATCATTTGCTCG	55.2	122
Reverse	GGGCATGGTTTCGTCTGG	62.5
*MHC*	Forward	AGGGAGCTTGAAAACGAGGT	60.2	260
Reverse	GCTTCCTCCAGCTCGTGCTG	65.9
*Tbp*	Forward	TGCACAGGAGCCAAGAGTGAA	64.4	132
Reverse	CACATCACAGCTCCCCACCA	65.6
*Gapdh*	Forward	ATGTTTGTGATGGGTGTGAA	57.2	151
Reverse	ATGCCAAAGTTGTCATGGAT	57.9
*Hprt*	Forward	ATGGACTGATTATGGACAGGACTG	61.5	124
Reverse	TCCAGCAGGTCAGCAAAGAAC	62.8
*Rps12*	Forward	AAGGCATAGCTGCTGGAGGTGTAA	65.3	156
Reverse	AGTTGGATGCGAGCACACACAGAT	67.4

∗ bp = base pairs.

**Supplementary Table II:** Optimized LC MS/MS parameters used for analysis of statins

Compound	Precursor type	Parent ion m/z	Daughter ion m/z	Capillary voltage (kV)	Cone voltage (V)	Collision energy (eV)
SVL	[M+H]^+^	419.29	119.17	4.00	35	23
SVA	[M-H]^−^	435.49	319.43	3.20	45	14
PVL	[M+H]^+^	407.46	183.22	3.5	35	18
PVA	[M-H]^−^	423.23	321.37	3.2	35	16
LOV-L	[M+Na]^+^	427.15	325.36	4.00	35	23
LOV-A	[M-H]^−^	421.08	319.54	3.20	45	14

*Abbreviations: LOV-A*, lovastatin acid; *LOV-L*, lovastatin lactone; *PVA*, pravastatin hydroxy acid; *PVL*, pravastatin lactone; *SVA*, simvastatin hydroxy acid; *SVL*, simvastatin lactone.

**SupplemenItary Table III:** Primers used for gene expression quantification of cellular transporters

Gene	Direction	Primer sequence (5′-3′)	Melting temperature (°C)	Amplicon length (bp)∗
*MRP1*	Forward	GTTATGGAAGCCTGTGCCCT	60.03	296
Reverse	ATGACATCCACTTGGGGCAG	60.03
*MRP4*	Forward	CCTGGTGAGTTGTTAGCCGT	59.97	326
Reverse	AGGTTCACCCGAGCTTTCTG	59.96
*MRP5*	Forward	CTTCCTGGGATCAGCGGTTT	60.04	244
Reverse	GCTCTGAAAGTACCCGGCTT	60.04
*OATP1a4*	Forward	AACTCCCATCATGCCCTTGG	60.03	246
Reverse	CCTGCACAGACCAAAAAGCC	59.97
*OATP2b1*	Forward	GCCAGAAGGAGGCATCAACT	60.03	233
Reverse	TTAAAGGCTCGTGCTGGGAG	60.04
*OATP1a4*	Forward	CTCACTTGGCTGTGATTCTTGT	58.86	254
Reverse	TCTTCTGATTGTCTCCAAATGTCT	57.88
*OATP2b1*	Forward	TCATCCTGAGAGGTGTGAAGAAAG	60.02	203
Reverse	TATGAATCGGTTTCGGAGCAGG	60.48
*OATP1a4*	Forward	CCACGTCTGTAGTTGGGCTT	59.97	291
Reverse	TCACACACTCTGTTGGGTCT	58.50
*OATP2b1*	Forward	TGCCAGAAGGAGGCATCAAC	60.32	346
Reverse	ACCAGCAAGAAGATGGGGTG	59.96
*Gapdh*	Forward	TGTGAACGGATTTGGCCGTA	59.96	159
Reverse	ACTGTGCCGTTGAATTTGCC	59.97
*Hprt*	Forward	AGTCCCAGCGTCGTGATTAG	59.54	170
Reverse	TGATGGCCTCCCATCTCCTT	60.33
*Rps12*	Forward	ACAAGAGGTGCTGAAGACCG	59.97	275
Reverse	CTACGCAACTGCAACCAACC	60.04
*Tbp*	Forward	CTCAGTTACAGGTGGCAGCA	59.96	118
Reverse	GCACAGAGCAAGCAACTCAC	60.04

∗ bp = base pairs.

**Supplementary Table IV:** Intraday and interday precision and accuracy of HPLC methods used for determination of statins in human plasma samples. Both intraday and interday assay variability values were within acceptance criteria for all tested compounds at the 4 QC levels

Statins	Quality control levels		Intraday (n = 6)		Interday (n = 6)	
Concentration (μmol/L)	Concentration (μg/mL)	Precision (RSD%)∗	Accuracy (RE%)†	Precision (RSD%)	Accuracy (RE%)
Simvastatin lactone	LLOQ (0.156)	0.065	9.70	−5.94	14.56	−3.05
LQC (5)	2.09	3.74	−10.13	14.79	−3.12
MQC (60)	25.12	12.43	0.57	14.30	0.50
HQC (120)	50.23	9.78	−1.36	14.44	5.90
Simvastatin hydroxy acid	LLOQ (0.313)	0.142	11.61	9.98	11.58	17.42
LQC (5)	2.26	14.83	0.37	10.91	−1.33
MQC (60)	27.21	6.54	−6.14	6.43	−2.45
HQC (120)	54.43	6.72	−11.70	7.69	−8.93
Pravastatin lactone	LLOQ (0.234)	0.095	3.33	13.88	6.95	14.65
LQC (10)	4.06	2.38	−3.17	6.99	−9.67
MQC (100)	40.65	12.46	2.34	11.74	5.21
HQC (200)	81.30	4.43	6.91	7.27	8.16
Pravastatin hydroxy acid	LLOQ (0.469)	0.209	17.39	−9.86	16.02	−4.53
LQC (10)	4.46	8.35	12.51	8.17	11.50
MQC (100)	44.65	11.84	−10.22	13.45	−10.71
HQC (200)	89.30	9.85	−12.94	10.46	−11.32

*Abbreviations:HQC*, high concentration quality control; *LLOQ*, lower limit of quantification; *LQC*, low concentration quality control; *MQC*, medium concentration quality control.

∗ Relative standard deviation (RSD%) = (standard deviation [SD]/mean of obtained concentration) × 100.

† Relative error (RE%) = ([measured concentration−nominal concentration]/nominal concentration) × 100.

**Supplementary Table V:** Intraday and interday precision and accuracy of HPLC methods used for determination statins in PBS samples. Both intraday and interday assay variability values were within acceptance criteria for all tested compounds at the 4 QC levels

Statins	Quality control levels		Intraday (n = 6)		Interday (n = 6)	
Concentration (μmol/L)	Concentration (μg/mL)	Precision (RSD%)∗	Accuracy (RE%)†	Precision (RSD%)	Accuracy (RE%)
Simvastatin lactone	LLOQ (0.156)	0.065	1.36	−7.04	7.91	15.80
LQC (5)	2.09	4.51	−0.81	3.92	−11.04
MQC (60)	25.12	11.89	−8.30	13.49	9.62
HQC (120)	50.23	10.39	−6.69	10.16	−8.67
Simvastatin hydroxy acid	LLOQ (0.313)	0.142	3.75	−6.69	11.60	−19.38
LQC (5)	2.26	8.39	−13.58	13.55	6.56
MQC (60)	27.21	12.01	9.41	9.88	6.14
HQC (120)	54.43	9.65	−1.8	10.75	−0.53
	LLOQ (0.234)	0.095	4.16	7.06	19.92	−2.66
Pravastatin lactone	LQC (10)	4.06	2.45	9.66	2.71	14.11
MQC (100)	40.65	5.92	9.35	7.05	−3.10
HQC (200)	81.30	5.28	14.57	6.18	−1.46
Pravastatin hydroxy acid	LLOQ (0.469)	0.209	2.12	19.99	14.32	−15.86
LQC (10)	4.46	3.52	8.81	8.39	13.08
MQC (100)	44.65	4.50	7.56	4.86	1.79
HQC (200)	89.30	12.69	4.79	4.93	2.29

*Abbreviations:HQC*, high concentration quality control; *LLOQ*, lower limit of quantification; *LQC*, low concentration quality control; *MQC*, medium concentration quality control; *PBS*, phosphate buffer saline.

∗ Relative standard deviation (RSD%) = (standard deviation [SD]/mean of obtained concentration) × 100.

† Relative error (RE%) = ([measured concentration−nominal concentration]/nominal concentration) × 100.

**Supplementary Table VI:** Intraday and interday precision and accuracy of HPLC methods used for determination of statins in DMEM culture medium samples. Both intraday and interday assay variability values were within acceptance criteria for all tested compounds at the 4 QC levels

Statins	Quality control levels		Intraday (n = 6)		Interday (n = 6)	
Concentration (μmol/L)	Concentration (μg/mL)	Precision (RSD%)∗	Accuracy (RE%)†	Precision (RSD%)	Accuracy (RE%)
Simvastatin lactone	LLOQ (0.156)	0.065	2.06	7.36	17.28	−3.25
LQC (5)	2.09	10.13	6.83	11.82	3.8
MQC (40)	16.74	2.36	−3.34	9.35	11.15
HQC (120)	50.23	6.71	−2.71	10.13	0.41
Simvastatin hydroxy acid	LLOQ (0.313)	0.142	3.99	−18.51	11.48	−15.85
LQC (5)	2.26	11.97	6.67	12.7	1.97
MQC (40)	18.14	11.47	−12.34	9.44	−14.98
HQC (120)	54.43	2.68	−5.3	3.54	−4.16
Pravastatin lactone	LLOQ (0.234)	0.095	8.14	17.37	8.87	18.54
LQC (10)	4.06	7.87	6.59	4.07	12.59
MQC (100)	40.65	1.6	−6.71	1.5	−4.67
HQC (200)	81.30	3.63	−5.66	4.2	−2.74
Pravastatin hydroxy acid	LLOQ (0.469)	0.209	7.28	−3.05	18.19	−8.12
LQC (10)	4.46	3.42	−4.19	1.64	−7.99
MQC (100)	44.65	1.54	2.01	1.19	1.29
HQC (200)	89.30	7.13	−5.56	9.75	−3.37

*Abbreviations:DMEM*, Dulbecco's modified eagle medium; *HQC*, high concentration quality control; *LLOQ*, lower limit of quantification; *LQC*, low concentration quality control; *MQC*, medium concentration quality control.

∗ Relative standard deviation (RSD%) = (standard deviation [SD]/mean of obtained concentration) × 100.

† Relative error (RE%) = ([measured concentration−nominal concentration]/nominal concentration) × 100.

**Supplementary Table VII:** Intraday and interday precision and accuracy of LC MS/MS methods used for determination of statins in cell lysate samples. Both intraday and interday assay variability values were within acceptance criteria for all tested compounds at the 4 QC levels

Statins	Quality control levels		Intraday (n = 6)		Interday (n = 6)	
Concentration (nmol/L)	Concentration ng/mL	Precision (RSD%)∗	Accuracy (RE%)†	Precision (RSD%)	Accuracy (RE%)
Simvastatin lactone	LLOQ (5)	2.09	14.34	10.06	15.89	9.60
LQC (20)	8.37	12.35	7.44	13.70	−1.79
MQC (200)	83.72	12.91	5.47	10.71	9.24
HQC (2000)	837.17	12.45	8.30	10.52	3.93
Simvastatin hydroxy acid	LLOQ (2.5)	1.13	7.215	−3.17	9.30	0.16
LQC (20)	9.07	4.69	8.75	5.18	9.52
MQC (200)	90.72	5.12	3.51	6.59	4.77
HQC (2000)	907.24	8.38	−5.44	8.19	−7.57
Pravastatin lactone	LLOQ (5)	2.03	9.98	14.16	6.96	10.00
LQC (20)	8.13	11.46	−4.73	11.64	−2.20
MQC (200)	81.3	11.36	0.03	9.43	3.72
HQC (2000)	813	7.3	−0.20	6.28	−0.11
Pravastatin hydroxy acid	LLOQ (5)	2.23	17.04	7.30	12.93	11.62
LQC (20)	8.93	11.76	−6.32	12.38	−6.87
MQC (200)	89.30	12.28	−9.45	12.01	−3.61
HQC (2000)	893.02	11.10	0.08	8.79	0.03

*Abbreviations:HQC*, high concentration quality control; *LLOQ*, lower limit of quantification; *LQC*, low concentration quality control; *MQC*, medium concentration quality control.

∗ Relative standard deviation (RSD%) = (standard deviation [SD]/mean of obtained concentration) × 100.

† Relative error (RE%) = ([measured concentration−nominal concentration]/nominal concentration) × 100.

**Supplementary Table VIII:** Extraction recovery (%) of statins from human plasma, PBS, and DMEM samples

Statins	Quality control levels	% Of statin recovered (mean ± SD, n = 6)		
Plasma samples	PBS samples	DMEM samples
Simvastatin lactone	LQC	80.75 ± 14.89	96.80 ± 6.04	94.48 ± 2.76
MQC	89.14 ± 7.40	96.28 ± 11.12	93.41 ± 2.94
HQC	87.52 ± 13.50	92.94 ± 12.23	82.68 ± 7.48
Simvastatin hydroxy acid	LQC	87.61 ± 16.11	90.15 ± 12.3	94.43 ± 3.77
MQC	84.61 ± 15.74	86.66 ± 14.98	70.82 ± 6.81
HQC	88.13 ± 10.66	95.57 ± 3.32	83.40 ± 20.31
Pravastatin lactone	LQC	76.53 ± 16.27	88.52 ± 9.11	78.81 ± 26.59
MQC	85.40 ± 16.22	86.55 ± 3.98	91.46 ± 13.07
HQC	80.58 ± 8.47	88.43 ± 8.37	90.11 ± 12.15
Pravastatin hydroxy acid	LQC	78.56 ± 7.80	77.32 ± 5.06	77.83 ± 11.90
MQC	83.08 ± 13.27	81.71 ± 26.71	78.73 ± 5.33
HQC	81.95 ± 15.63	88.73 ± 31.67	88.48 ± 5.36

*Abbreviations:DMEM*, Dulbecco's modified eagle medium; *HQC*, high concentration quality control; *LLOQ*, lower limit of quantification; *LQC*, low concentration quality control; *MQC*, medium concentration quality control; *PBS*, phosphate buffer saline.

**Table I:** The pH dependence of the hydrolysis of the lactone form of statins

Sample pH	Simvastatin lactone (half-life, hours)			Pravastatin lactone (half-life, hours)		
	PBS	Human plasma	DMEM	PBS	Human plasma	DMEM
6.8	26.68 ± 0.97	36.51 ± 4.37∗^,^†	21.42 ± 2.35∗	21.22 ± 1.75	8.84 ± 0.31∗^,^†	17.30 ± 4‡
7.0	16.92 ± 0.86	21.30 ± 2.50∗	-	12.96 ± 1.38	7.39 ± 0.22	-
7.2	13.68 ± 0.71	14.46 ± 3.55	11.70 ± 0.50	9.36 ± 1.57	6.61 ± 0.95§^,^†	11.89 ± 2.71
7.4	12.86 ± 0.31	10.45 ± 0.38	7.40 ± 0.37∗	6.89 ± 0.41	6.85 ± 0.54‖	9.72 ± 1.42§
7.6	9.58 ± 1.67	9.16 ± 1.77	-	5.59 ± 0.49	NA	-
7.8	10.23 ± 1.18	8.61 ± 3.38	7.09 ± 0.30	4.98 ± 0.44	NA	10.66 ± 0.81‡

*Abbreviations: DMEM*, Dulbecco's modified eagle medium; *NA*, nonapplicable; *PBS*, phosphate buffer saline.

Data are presented as mean ± SD of 6 replicates. Statistical analysis was done by 2-way analysis of variance followed by Bonferroni's post hoc test.

∗ Significant difference from PBS samples (*P* < 0.001).

† Significant difference from DMEM samples (*P* < 0.001).

‡ Significant difference from PBS samples (*P* < 0.01).

§ Significant difference from DMEM samples (*P* < 0.05).

‖ Significant difference from DMEM (*P* < 0.05).

**Table II:** Cytotoxicity of statins to C2C12 cells after 72 hours of treatment in medium of different pH. Data are presented as mean ± SD of 3 experiments, 8 replicates per experiment

Statin	C2C12 cells	IC_50_ values of statins (μmol/L)		
Acidic medium	Neutral medium	Alkaline medium
Simvastatin lactone	Undifferentiated cells	0.44 ± 0.02∗	0.58 ± 0.02†	1.13 ± 0.04‡
Differentiated cells	15.79 ± 0.77∗^,^§	40.76 ± 1.75†^,^§	56.99 ± 1.86‡^,^§
Simvastatin hydroxy acid	Undifferentiated cells	2.22 ± 0.04∗	4.35 ± 0.09†	16.85 ± 0.48‡
Differentiated cells	65.41 ± 2.69§	NA	NA
Pravastatin lactone	Undifferentiated cells	85.59 ± 2.05	NA	NA
Differentiated cells	109.9 ± 3.74§	NA	NA

*Abbreviations: NA*, non-applicable; *SD*, standard deviation.

Data are presented as mean ± SD of 3 experiments, 8 replicates per experiment. Statistical analysis was done by 2-way analysis of variance followed by Bonferroni's post hoc test.

∗ Significant difference from neutral and alkaline medium (*P* < 0.001).

† Significant difference from acidic and alkaline medium (*P* < 0.001).

‡ Significant difference from acidic and neutral medium (*P* < 0.001).

§ Significant difference from undifferentiated cells (*P* < 0.001).
